# On photokinetics under monochromatic light

**DOI:** 10.3389/fchem.2023.1233151

**Published:** 2023-09-14

**Authors:** Mounir Maafi

**Affiliations:** Leicester School of Pharmacy, De Montfort University, Leicester, United Kingdom

**Keywords:** Φ-order kinetics, photokinetics, quantum yield, actinometry, solving kinetics, monochromatic light

## Abstract

The properties of photokinetics under monochromatic light have not yet been fully described in the literature. In addition, for the last 120 years or so, explicit, handy model equations that can map out the kinetic behaviour of photoreactions have been lacking. These gaps in the knowledge are addressed in the present paper. Several general features of such photokinetics were investigated, including the effects of initial reactant concentration, the presence of spectator molecules, and radiation intensity. A unique equation, standing for a pseudo-integrated rate law, capable of outlining the kinetic behaviour of any photoreaction is proposed. In addition, a method that solves for quantum yields and absorption coefficients of all species of a given photoreaction is detailed. A metric (the initial velocity) has been adopted, and its reliability for the quantification of several effects was proven by theoretical derivation, Runge–Kutta numerical integration calculations and through the model equation proposed. Overall, this study shows that, under monochromatic light, photoreaction kinetics is well described by Φ -order kinetics, which is embodied by a unifying model equation. This paper is aimed at contributing to rationalising photokinetics via reliable, easy-to-use mathematical tools.

## 1 Introduction

Light-sensitive chemicals are ubiquitous in nature and increasingly becoming a part of new or potential technological and industrial processes. From a general application point of view, two main themes stir the interest in such molecular systems and materials: relating to their direct potential benefit and/or to avoid their degradation. For instance, photolabile molecular systems find applications in nanotechnology (Shit et al., 2020), biomimetic chemistry (Cahova et al., 2013), optical information storage (Irie, 2015; Nakatani et al., 2016; Sarter, et al., 2016; Pianowski, 2022), surface science (Bonacchi et al., 2015), environmental chemistry (Nakatani et al., 2016; Malato et al., 2021), photo-responsive materials (Irie, 2000; Irie et al., 2014; Okuda et al., 2016), photoswitchable nucleosides (Singer and Jaschke, 2010; Cahova and Jaschke, 2013; Singer et al., 2013; Chen et al., 2014), pharmaceutical drugs (Velema et al., 2014), photoenergy (Montalti et al., 2020), and green chemistry (Scaiano, 2020; Pianowski, 2022), to cite a few fields. The trend of such applications is expected to grow in the future (Bonfield et al., 2020).

One of the primary tools for the study of molecular reactions is chemical kinetics, specifically photokinetics in the case of photoreactions. Among its aims, photokinetics allows to unravel the intrinsic parameters (i.e., quantum yields and absorptivities) of the reaction at hand. Such information is necessary for the understanding of reaction behaviour and control. Unfortunately, the area of photokinetics remains significantly underdeveloped compared to (thermal) chemical kinetics. The former not only lacks the usual metrics used in chemical kinetics (such as the kinetic order of a reaction and/or integrated solutions of the rate laws) but also suffers from the absence of standard methods, a situation that might explain a considerable lack of text books on the subject, where, surprisingly, we only find a single entry that is fully dedicated to photokinetics (a textbook published in 1998) (Mauser and Gauglitz, 1998).

The main hindrance facing a normal development of photokinetics can be traced down to a mathematical impossibility to analytically solve the rate laws of photoreactions (Mauser et al., 1998; Crano and Guglielmetti, 2003; Tonnesen, 2004; Nakatani et al., 2016; Montalti et al., 2020; Scaiano, 2020; Pianowski, 2022). The absence of integrated rate laws has considerably limited photokinetic investigation towards unravelling photoreaction features, offering accessible metric tools to the experimentalist, and/or allowing better control of the reactions, as usually available for thermal kinetic studies.

In this context, several approaches have been usually adopted to circumvent this mathematical hurdle. In the first instance, one can notice the ubiquity of the usage of thermal kinetics’ equations (such as those corresponding to zeroth- and first-order kinetics) in the literature that deals with data of photoreactions (Crano and Guglielmetti, 2003; Tonnesen, 2004; Nakatani et al., 2016; Malato et al., 2021; Pianowski, 2022). Despite its popularity, this approach can be deemed inappropriate due to a significant difference between the mathematical formulations of the rate laws describing thermal and photochemical reactions.

A second, more elaborate method employs expansion with a power series of the rate-law terms involving power numbers, in order to achieve integration of the rate law. In general, this approach is applied to the simplest photoreactions (e.g., no known example has treated the photoreversible reaction) and limits the expansion to the first order of the power series. Even if an analytical solution can be achieved in this case, it still remains of limited application due to the stringent condition imposed on the allowed magnitude of the absorbance (which might be as low as 0.01). If expansion to a higher order of the power series is envisaged, in order to alleviate the absorbance limit and reach workable experimental values, the differential equation becomes, unfortunately, unsolvable analytically.

Till now, the best treatment of photokinetic data has been achieved by employing numerical integration methods (NIMs). NIMs are very powerful tools for this purpose, but their usage and technicalities are not necessarily familiar to most experimentalists in the field of photokinetics. More importantly, the efficacity of NIMs remains dependent on the number of unknown reaction parameters to define. In general, NIMs work fine when only a small number of parameters are sought but will face an identifiability problem (Maafi and Brown, 2005a) as the number of those parameters increases (for instance, NIMs are not capable of unravelling the true parameters for a photoreversible reaction if both forward and reverse quantum yields, as well as the absorption coefficient of the photoproduct, are unknown). We shall discuss this issue further in Section 3.5.

According to the aforementioned succinct review, photokinetics is still in need of both i) a simple way that should be accessible to all, allowing photokinetic data investigation, and ii) a standard and reliable set of model kinetic equations that allow consistent photokinetic studies and conclusions.

In our team, we have previously proven that the photokinetics of the primary photoprocess ( X → Y , whose photoproduct, Y , is transparent to the monochromatic irradiating beam) can be solved analytically and the reaction’s behaviour obeys Φ -order kinetics (Maafi and Brown, 2007). The analytically derived solution for this reaction provides both an integrated rate law involving a logarithmic function bearing a time-exponential term in its argument (*vide infra* Eq. 4) and an explicit formula for the rate constant of the photoreaction (Maafi and Brown, 2007). However, when the product of the primary photoprocess absorbs concomitantly with the reactant, there are two distinct kinetic cases: a) the rate law of the reaction becomes non-integrable if the monochromatic light wavelength is different from that of an isosbestic point (non-isosbestic irradiation) (Maafi and Maafi, 2013) and b) the reaction obeys the first-order kinetics when the irradiation light is isosbestic (Maafi and Brown, 2005b). It is interesting to notice that such a dual (a/b) kinetic pattern was preserved for all sub-mechanisms of a cyclic trimolecular system involving six photochemical steps (Maafi and Brown, 2005b).

Furthermore, a semi-empirical approach combining Runge–Kutta (RK) numerical integration trace data and an explicit $C\left(t\right)$ algebraic equation of the Φ -order type was proposed to overcome the latter mathematical hurdle, when irradiation is non-isosbestic, and deliver exploitable explicit models for other reactions than the primary photoreaction discussed previously. The aim was to achieve consistency and precision in describing the photokinetics and to determine both unknown quantum yields and absorption coefficients of the reaction at hand. The approach was developed and experimentally applied to the primary photoprocess with absorbing the reactant and product (Maafi and Maafi, 2013), the photoreversible reaction (Maafi and Maafi, 2014a; 2014b), and the multi-consecutive photoreaction involving four photoproducts (Maafi and Maafi, 2016). One of the interesting experimental results facilitated by those formulae was evidence of the variability of the quantum yield (of each individual reaction step) with the wavelength of the irradiation light, as has been shown for a variety of photoreactive molecules that belonged to different chemical families (Maafi and Brown, 2007; Maafi and Maafi, 2013; Maafi and Maafi, 2014b; Maafi and Lee, 2015a; Maafi and Lee, 2015b; Maafi and Maafi, 2016; Maafi and Al qarni, 2019; Maafi and Al qarni, 2022).

Even if the $C\left(t\right)$ explicit expressions proposed for the latter reactions were very useful, and the semi-empirical method can, in principle, be developed/applied to any photoreaction, it is, however, a fact that the semi-empirical equations are, thus far, only available for a handful of reactions, and they are restricted to absorbance limits.

Nonetheless, our results have proven that using explicit formulae is not only handy but also provides a better assessment of reaction photokinetics. However, there is a gap in the knowledge that needs to be addressed. From this perspective, it would be an advantage to have a model equation that fits all reactions without imposing any constraints on the initial concentrations or absorbance. It would also be of great interest to have a methodology and metrics to assess the reaction, define its unknown quantum yields and absorption coefficients, evaluate the quantum yield variation with wavelength, and develop new actinometers. The present study is dedicated to an attempt to bring adequate answers to the aforementioned points for photoreactions performed under monochromatic light. In addition, it proposes a review of the main properties of the reaction considered in different situations. Such an overview has not been presented, thus far, in photochemistry and kinetic literature.

## 2 Experimental

**SCHEME 1:**
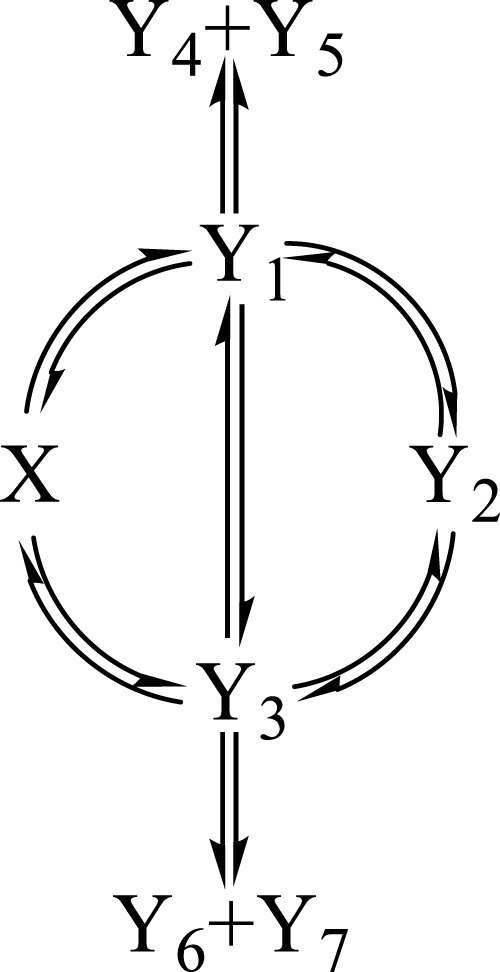
Φ -shaped photomechanism involving eight reactive species interlinked by 14 photochemical reaction steps.

Each tested reaction mechanism is identified by a reactant X , *j* photoproducts ( $Y_{j}$ ) (where $0\leq j\leq n_{sp}$ , with $X=Y_{0}$ , and $n_{sp}$ is the total number of species in the reaction medium), and $n_{\Phi j}$ photoreaction steps starting or ending at species $Y_{j}$ , according to the Φ-shaped mechanism given in Scheme 1.

Time evolution of concentrations corresponding to each species involved in the mechanism (kinetic traces $C_{X}\left(t\right)$ and $C_{Y_{j}}\left(t\right)$ ) was numerically calculated. The NIM selected for the present work consisted of a fourth-order RK NIM. The RK-calculated data were generated by a homemade programme. The code runs on an Excel VBA platform that is available on Microsoft Excel.

RK-calculated traces served as a reference for testing the performance of the proposed model formula (*vide infra* Eq. 4). The RK simulations were conducted at iteration intervals ranging between 0.1 and 5 s. The fitting of the RK-generated traces to Eq. 4 (for X and each $Y_{j}$ of a given mechanism) was performed with a Levenberg–Marquardt algorithm (LMA) provided by the curve-fitting tool of R2020b Matlab software. The goodness of fit of the traces was assessed by i) the values of the squared correlation coefficients (*r* ^2^), characterising the linear plot between the data of the RK-simulated traces and those supplied by the LMA-calculated curves based on the respective Eq. 4 of the reactant and each species $Y_{j}$ of the reactive system, ii) the sum of squares error (SSE), and iii) the root mean square deviation (RMSD) between the two datasets. The parameters of Eq. 4 ( $w_{ij}$ , ${cc}_{j}$ , and $k_{ij}$ ) were obtained by LMA within 95% confidence limits.

The number of mono- Φ -order terms (i.e., $\omega \;\mathrm{Log}\left(1+cc\;e^{-kt}\right)$ ) in each Eq. 4, describing each of the traces of the species involved in the reaction mechanism at hand, cannot exceed the maximum value of $n_{\Phi }$ , but it can be lower than $n_{\Phi }$ (i.e., $i_{j}$ ), depending on the trace. The RK calculations assume *de facto* that the concentrations of the reactant and the $Y_{j}$ species belong to the respective linearity ranges of those species’ calibration graphs, throughout the duration of the reaction (irrespective of the reaction investigated) (it is to be noted that Eq. (1) is not valid for high concentrations that lay beyond the respective linearity ranges of the species).

The $n_{sp}$ fitting equations (of Eq. 4 type) corresponding to the reactant and *j* photoproducts (involved in a given reactive system) are effectively coupled in the sense that Eq. 4 of species $Y_{j}$ necessarily shares some, if not all, of its k values with Eq. 4 of previous species $Y_{j-1}$ (occurring in the reaction mechanism before species $Y_{j}$ ).

The rate laws considered in the present work apply to a slab-shaped, continuously and vigorously stirred reactor subjected to a collimated monochromatic light beam.

## 3 Results and discussion

### 3.1 The rate-law equation

As for thermal kinetics, in photokinetics, a rate law should be written for each species $Y_{j}$ (where 0≤j≤7 , with the reactant X= $Y_{0}$ and the photoproducts $Y_{1\;to\;7}$ ) of the reaction mechanism (Scheme 1). The $n_{\Phi j}$ reaction steps linking $Y_{j}$ can correspond to either forward or backward reactions with other $Y_{j^{\prime }}$ species ( $j\neq j^{\prime }$ ). Each rate law, for a given $Y_{j}$ ( $r_{Y_{j}}^{\lambda_{irr}}\left(t\right)$ ), can then take the following general formulation, irrespective of the actual reaction mechanism.

$$r_{Y_{j}}^{\lambda_{irr}}\left(t\right)={\left(C_{Y_{j}}^{\lambda_{irr}}\left(t\right)\right)}^{\prime }=\sum_{j^{\prime }\neq j}^{n_{\Phi j}}-\Phi_{Y_{j}\;\to \;Y_{j^{\prime }}}^{\lambda_{irr}}\;P_{a_{Y_{j}}}^{\lambda_{irr}}\left(t\right)+\Phi_{Y_{j^{\prime }}\;\to Y_{j}}^{\lambda_{irr}}\;P_{a_{Y_{j^{\prime }}}}^{\lambda_{irr}}\left(t\right).$$ (1)

It is to be noted that it is unusual in kinetics that the concentration of species $Y_{j}$ at time t is written relative to the irradiation wavelength, as given in Eq. (1). This labelling is necessary simply because all the parameters on the right-hand side of Eq. (1) are dependent on $\lambda_{irr}$ . This makes two traces of a species, obtained at two different wavelengths $\lambda_{irr_{1}}$ and $\lambda_{irr_{2}}$ (e.g., $C_{Y_{j}}^{\lambda_{irr_{1}}}\left(t\right)$ and $C_{Y_{j}}^{\lambda_{irr_{2}}}$ ), most certainly different from each other.

In Eq. (1), the light absorbed by species $Y_{j}$ or $Y_{j^{\prime }}$ ( $P_{a_{Y_{j}}}^{\lambda_{irr}}$ and $P_{a_{Y_{j^{\prime }}}}^{\lambda_{irr}}$ , with $j\neq j^{\prime }$ ), constitutes fractions of the total absorbed light by the medium ( $P_{a}^{\lambda_{irr}}$ ) at time t (expressed in $einstein\;s^{-1}\;{dm}^{-3}$ ), according to the following expression:

$$P_{a_{Y_{j\;or\;j^{\prime }}}}^{\lambda_{irr}}\left(t\right)=\frac{A_{Y_{j\;or\;j^{\prime }}}^{\lambda_{irr}}\left(t\right)}{A_{tot}^{\lambda_{irr}}\left(t\right)}\;P_{0}^{\lambda_{irr}}\;\left(1-10^{-A_{tot}^{\lambda_{irr}}\left(t\right)}\right)=A_{Y_{j\;or\;j^{\prime }}}^{\lambda_{irr}}\left(t\right)\;P_{0}^{\lambda_{irr}}\;PKF\left(t\right),$$ (2)

where $P_{0}^{\lambda_{irr}}$ is the intensity of the incident radiation (see Section 3.9 for a detailed definition), the total absorbance ( $A_{tot}^{\lambda_{irr}}$ ) is a sum of the individual absorbances of the species ( $A_{Y_{j}}^{\lambda_{irr}}$ ) at $\lambda_{irr}$ ( $n_{sp}$ , in Eq. (3), represents the number of all species $Y_{j}$ —reactant and products—in the medium at time t ), and $PKF\left(t\right)$ is the dimensionless photokinetic factor.

$$A_{tot}^{\lambda_{irr}}\left(t\right)=\sum_{j=0}^{n_{sp}}A_{Y_{j}}^{\lambda_{irr}}\left(t\right).$$ (3)

Obtaining analytical solutions of Eq. (1) rate-law equations, for a vast majority of cases, is simply impossible. This mathematical hurdle can be explained by the fact that these differential equations are non-linear due to the presence of the time-variable term $\left(1-10^{-A_{tot}^{\lambda_{irr}}}\right)$ , which encompasses all absorbances of the species present in the reactive medium. This situation is expected to persist for the foreseeable future until a mathematical technique, capable of analytically solving these complicated differential equations, is devised.

### 3.2 The proposed integrated rate-law model

The traces of a primary photoprocess subjected to monochromatic light were proven to obey a Φ -order equation that was analytically derived from the reaction’s rate law (Maafi and Brown, 2007). Previous work on other photomechanisms (Maafi and Maafi, 2013; Maafi and Maafi, 2014a; Maafi and Maafi, 2016), whose rate laws cannot be solved by closed-form integrations, has also shown that the traces of photoreactions, involving up to five photochemical steps, can be well described by specific semi-empirical formulae, which mathematically possess a Φ -order character, but under certain constraints on the total absorbance at the end of the reaction ( $A_{tot}^{\lambda_{irr}}\left(\infty \right)$ ranging between 0.2 and 0.6). However, till now, no general formula has been proposed for the description of traces of photoreactions. A conjecture, based on the aforementioned results, might suggest that such a general equation would be of a Φ -order type.

Accordingly, the following explicit formula (Eq. 4) is proposed to describe the trace of species $Y_{j}$ ( $C_{Y_{j}}^{\lambda_{irr}}\left(t\right)$ , j=0 for the reactant X ), irrespective of the mechanism undergone by the actual photoreaction, and the wavelength ( $\lambda_{irr}$ ) of the non-isosbestic monochromatic irradiation light driving the reaction. The reaction mechanism may involve any number of photochemical reaction steps (e.g., within Scheme 1). The temporal variation in the concentration of a typical species $Y_{j}$ is given by Eq. 4 (where Log and e are, respectively, the base 10 logarithm and the exponential functions and $i_{j}$ is the number of mono- Φ -order terms under the sum).

$$C_{Y_{j}}^{\lambda_{irr}}\left(t\right)=C_{\infty ,j}^{\lambda_{irr}}+\sum_{i=1}^{i_{j}}\omega_{ij}^{\lambda_{irr}}\;\mathrm{Log}\left(1+{cc}_{j}^{\lambda_{irr}}e^{-k_{ij}^{\lambda_{irr}}\;t}\right).$$ (4)

The expression for the reaction rate of species $Y_{j}$ is obtained by the differentiation of the corresponding Eq. 4 as

$${\left(C_{Y_{j}}^{\lambda_{irr}}\left(t\right)\right)}^{\prime }=r_{Y_{j}}^{\lambda_{irr}}\left(t\right)=-\sum_{i=1}^{i_{j}}\frac{\omega_{ij}^{\lambda_{irr}}\;{cc}_{j}^{\lambda_{irr}}\;k_{ij}^{\lambda_{irr}}\;e^{-k_{ij}^{\lambda_{irr}}\;t}}{\left(1+{cc}_{j}^{\lambda_{irr}}\;e^{-k_{ij}^{\lambda_{irr}}\;t}\right)\;\ln\left(10\right)},$$ (5)

from which we can derive the formula for $Y_{j}$ initial reaction rates (expressed in $M\;s^{-1}$ ) as

$${\left(C_{Y_{j}}^{\lambda_{irr}}\left(t\right)\right)}_{t=0}^{\prime }=r_{0Y_{j}}^{\lambda_{irr}}=-\frac{1}{\ln\left(10\right)}\;\sum_{i=1}^{i_{j}}\omega_{ij}^{\lambda_{irr}}\;k_{ij}^{\lambda_{irr}}\frac{{cc}_{j}^{\lambda_{irr}}}{1+{cc}_{j}^{\lambda_{irr}}}.$$ (6)

The parameters ( $C_{\infty ,j}^{\lambda_{irr}}$ , $\omega_{ij}^{\lambda_{irr}}$ , ${cc}_{j}^{\lambda_{irr}}$ , and $k_{ij}^{\lambda_{irr}}$ ) of Eq. 4 are specific to the irradiation wavelength ( $\lambda_{irr}$ ), making $C_{Y_{j}}^{\lambda_{irr}}$ wavelength specific. The parameter $C_{\infty ,j}^{\lambda_{irr}}$ , standing for the final concentration of species $Y_{j}$ , will be non-zero, but positive, if the species persists at the end of the reaction (typically for photostationary reactions and end products). The pre-exponential coefficient ${cc}_{j}^{\lambda_{irr}}$ is considered here to be the same for all $i_{j}$ terms making up Eq. 4 of a given species $Y_{j}$ ( ${cc}_{j}^{\lambda_{irr}}={cc}^{\lambda_{irr}}$ , a constraint that is verified practically). The pre-logarithmic parameter $\omega_{ij}^{\lambda_{irr}}$ , accepting positive and negative values, might be considered a weighing factor for the logarithm of the $i^{th}$ kinetic regime of the considered species $Y_{j}$ . Parameter $k_{ij}^{\lambda_{irr}}$ is the positive rate constant of the $i^{th}$ kinetic regime (expressed in $s^{-1}$ ) and is proportional to the light intensity and the quantum yield of the $i^{th}$ kinetic regime, but it may also depend on other attributes of the reactive species (these details are currently unknown).

### 3.3 The mechanism selected

In order to test the proposed equations (e.g., Eq. 4), a Φ -shaped mechanism is selected (Scheme 1, involving the reactant, X , seven photoproducts, Y , and 14 reaction steps). It is thought to be representative of a number of simple and complicated photoreactions encountered in photochemistry books and literature (Mauser et al., 1998; Crano and Guglielmetti, 2003; Tonnesen, 2004; Nakatani et al., 2016; Montalti et al., 2020; Scaiano, 2020; Pianowski, 2022). The number of sub-mechanisms that can be worked out from Scheme 1 exceeds 100 examplars (including the counting of the absorption of terminal species that might equal to zero).

The set of sub-mechanisms worked out from Scheme 1 includes, but is not limited to, those of the primary photoprocess with a transparent photoproduct (as for photochromic diarylethenes under visible light (Crano and Guglielmetti, 2003; Nakatani et al., 2016; Maafi and Alqarni, 2022; Pianowski, 2022)), the primary photoprocess with an absorbing photoproduct (as for nifedipine, nisoldipine, and dacarbazine drugs (Tonnesen, 2004; Maafi and Maafi, 2013; Maafi and Lee, 2015a)), the photoreversible reaction (as for stilbenoids (Crano and Guglielmetti, 2003; Maafi and Al-Qarni, 2019), anti-cancer drugs (Tonnesen, 2004; Maafi and Lee, 2015b), and dimethylhydropropenes (Ziani et al., 2021)), the consecutive double photoreversible system (as for fulgides in solution or in the solid state (Crano and Guglielmetti, 2003; Weerasekara et al., 2017; Kochman et al., 2022; Pianowski, 2022) and benzopyrans (Frigoli et al., 2020)), the multi-consecutive photoreaction (as for riboflavin (Maafi and Maafi, 2016)), and other more complex systems (Nakatani et al., 2016; Scaiano, 2020; Malato et al., 2021; Pianowski, 2022). The mechanisms and molecular systems reviewed previously have been extensively investigated, but their photokinetic studies tended to be either performed by using the equations of thermal kinetics (e.g., first-order exponential models), using various proposed (non-analytical) equations, or employing numerical integration. It is to be noted that no standard equation model has ever been provided in the aforementioned documentation and no integrated rate law has ever been analytically derived for photoreactions (including the simplest ones, such as the concurrent $Y_{3}\leftarrow X\to Y_{1}$ reaction).

### 3.4 Some general aspects of photo- and thermal kinetics

From a kinetic view point, the rate of a thermal reaction of a species $Y_{j}$ does not depend on the properties (e.g., concentrations) of distant species from $Y_{j}$ (that are not generated by or generate themselves $Y_{j}$ ), whereas in photokinetics, reactions’ rates depend not only on the properties of the species involved in the considered reaction step, as in thermal kinetics, but also on those of all the absorbing species occurring in the reaction medium over time. In fact, the rate of a photochemical reaction depends explicitly on the absorbances of all the species with a non-zero absorbance at a given time t . This particular difference between photo- and thermal kinetics is expressed in the rate equation (Eqs 1, 2) by the total absorbance of the set of reactive species ( $A_{tot}^{\lambda_{irr}}\left(t\right)$ ) present in the medium at a given time. Such a subtle interdependence of the reaction species within the photokinetic rate law is responsible for the species’ competition for the incident light. Therefore, species that belong to completely separated reaction steps of the overall reaction mechanism still influence each other’s reactivities. In the same context, the kinetics of species $Y_{j}$ might also be dependent on the absorbance of one or more inert (non-reactive) species, present in the medium as spectators, when the latter absorb at the irradiation wavelength. This particular phenomenon is further described in Section 3.7.

Furthermore, since the coefficients of the rate-law equation correspond to the attributes of the species and light at a given wavelength, it is evident that the solution of that equation is also specific to that wavelength. Therefore, changing the irradiation wavelength will cause the trace to change accordingly. This imposes that all Eq. 4 coefficients are indexed by $\lambda_{irr}$ (including the concentrations, $C_{Y_{j}}^{\lambda_{irr}}\left(t\right)$ ). In thermal kinetics, this would be equivalent to a change in temperature. Here, apart from a qualitative comparison (e.g., reaction speeds up between irradiations at $\lambda_{irr1}$ and $\lambda_{irr2}$ ), it is difficult to proceed with further quantitative analysis based on the comparison of the traces in the absence of an integrated rate law and the quantitative parameters it provides. For instance, the knowledge of the relative reaction velocity at a given time but at different wavelengths can help selecting the light wavelength range causing the species at hand to be most or least reactive depending on the purpose of the experimentalist/experiment. For some molecules (drugs, photochromes, etc.), it is it useful to specify the regions of light inducing most photoconversion, a situation which would be solved for photoreactions using Eq. 4, as will be shown in the following sections. One additional difference between thermal and photo-kinetics concerns an evidenced change in the photoreaction kinetic order. It was previously proven that the trimolecular cyclic photoreaction (Maafi and Brown, 2005b) and all its sub-mechanisms obeyed a first-order kinetics when irradiated with an isosbestic monochromatic beam ( $\lambda_{isos}$ coincides with an isosbestic point on overlayed successive absorption spectra of the medium through reaction time). It is reasonable to predict that any photoreaction will also be described by the first order kinetics whenever subjected to an isosbestic irradiation since the photokinetic factor [ $PKF\left(t\right)$ , Eq. (2)], under these conditions, is independent of time (which converts the rate law into a first-order linear differential equation). However, the kinetics of any photoreaction, under non-isosbestic light, extracted from the Φ-shaped mechanism, is shown in the following sections to follow Φ -order kinetics. The conjecture is to observe similar conclusions for any photoreaction. Accordingly, a general statement can be derived from the aforementioned observations: the kinetic data of a photoreaction can either obey a first-order or a Φ -order kinetics, respectively, when driven by an isosbestic or a non-isosbestic irradiation light (when all other experimental and reaction attributes are kept the same). A true change in the kinetic order then specifically occurs for photoreactions (the equivalent for thermal reactions would perhaps correspond to a change in the kinetic order when temperature changes, which is not observed). Also, it is noticeable that the mathematical formulation of the rate law explicitly includes a rate-constant factor (k) for thermal reactions but not for photochemical reactions.

Finally, the concentrations of the species involved in a photokinetic process must all belong to their respective calibration graphs (a redundant condition for thermal kinetics).

### 3.5 Total absorbance and fitting RK traces with the model equation

For generality purposes of the application of the model equation (Eq. 4), RK simulations were conducted on several sub-mechanisms derived from Scheme 1. Individual RK traces have been obtained for each species, involved in the sub-mechanism considered, and the fitting of each of these traces was performed with its corresponding Eq. 4.

**FIGURE 1:**
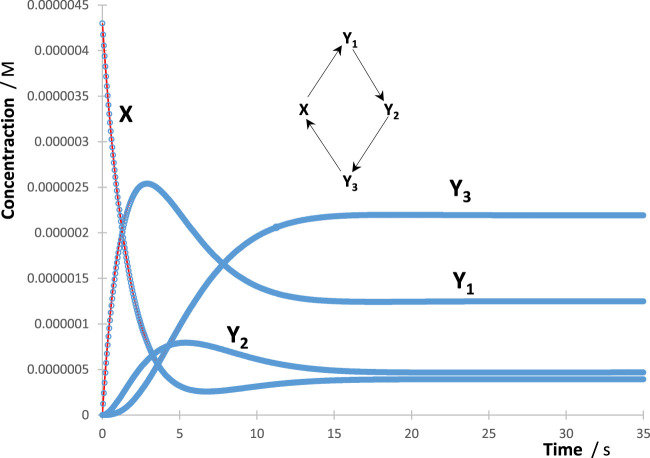
Example of a good fit of the reaction’s species RK traces (circles) by their corresponding Eqs. 4 (lines) for the indicated cyclic reaction mechanism. Each Eq. 4 of each of the species counted four mono- Φ -order terms, a unique set of four $k_{ij}^{\lambda_{irr}}$ values, and a single common value of ${cc}^{\lambda_{irr}}$ .

The process of RK trace fitting was implemented according to a simple protocol. The first trace to be fitted was the reactant’s, followed by that of the nearest $Y_{j}$ species in the reaction mechanism, then the next, and so on. The fitting parameters $k_{ij}^{\lambda_{irr}}$ obtained for an earlier species (e.g., $k_{10}^{\lambda_{irr}}$ and $k_{20}^{\lambda_{irr}}$ for X and j=0 ) were kept the same in the subsequent equations, describing the trace of the next species linked to X , where the latter might involve new terms (e.g., $k_{11}^{\lambda_{irr}}=k_{10}^{\lambda_{irr}}$ , $k_{21}^{\lambda_{irr}}=k_{20}^{\lambda_{irr}}$ , and $k_{31}^{\lambda_{irr}}$ for $Y_{1}$ ). Kinetically, this means that the trace of a given species in the reaction mechanism is necessarily dependent on the rate constants (both their number and values) of previous reaction steps generating that species in the reaction mechanism. Accordingly, the number $i_{j}$ of mono- Φ -order terms in Eq. 4 is minimal for the reactant and is expected to increase (or at least remains the same) for species occurring subsequently. The maximum number of terms in Eq. 4 that describe the trace of any single species cannot exceed the number of reaction steps occurring in the considered overall reaction mechanism ( $i_{j}\leq n_{\Phi }$ ). The minimum number of these terms should, in principle, be equal to the number of reaction steps starting or ending at the considered species (i.e., $n_{\Phi j}$ ). However, in some cases and depending on the kinetics, Eq. 4 can provide a good fit of the traces with less than $n_{\Phi j}$ terms. Interestingly, it is predicted that most likely the same number of terms is found for each Eq. 4 of each species in a cyclic reaction mechanism involving three or more molecules (e.g., Figure 1).

Furthermore, it has been observed that this fitting protocol indicates that factor ${cc}_{j}^{\lambda_{irr}}$ keeps the same value ( ${cc}_{j}^{\lambda_{irr}}={cc}^{\lambda_{irr}}$ ) for all Eq. 4 of all the species belonging to a given reaction mechanism. $C_{\infty ,j}^{\lambda_{irr}}$ takes the value of the final concentration of the considered species $Y_{j}$ , and $\omega_{ij}^{\lambda_{irr}}$ are different for different traces of the species of the same mechanism.

This coefficient, ${cc}^{\lambda_{irr}}$ , hence, plays the role of a coupling factor of the system of Eq. 4, describing the studied reaction. This feature will impose an additional constraint on the fitting of the photokinetic traces, which is expected to improve the reliability of the fitting parameters’ values.

It is possible to add a few more constraints to the fitting protocol, by considering Eq. 4 at the initial time ( t=0 ) and the known initial concentration values of the species.

$$C_{Y_{j}}^{\lambda_{irr}}\left(0\right)=C_{\infty ,j}^{\lambda_{irr}}+\sum_{i=1}^{i_{j}}\omega_{ij}^{\lambda_{irr}}\;\mathrm{Log}\left(1+{cc}_{j}^{\lambda_{irr}}\right).$$ (7)

In the present study, more than 200 RK traces were fitted according to the protocol described previously. These traces belonged to species involved in various sub-mechanisms derived from the Φ-shaped reaction (Scheme 1), including one to seven photoproducts and 1 to 14 reaction steps.

The individual species’ RK-generated traces were fit by adequate Eq. 4 of the reaction studied with various numbers of mono- Φ -order terms making up each of these equations. The protocol described previously for the treatment of the RK data with Eq. 4 worked extremely well for all traces. Excellent fittings of each individual trace (illustration in Figure 1) were obtained with correlation coefficient values, for RK-calculated *vs*. Eq. 4 data, not lower than 0.999, sums of squared errors (SSE) as low as 10^−22^, and root mean square errors (RMSE) of no higher than 10^−9^.

These results prove that the Φ -order kinetic behaviour is preserved for photochemical reactions, irrespective of the operating sub-mechanism that can be extracted from Scheme 1. This stands for a confirmation of our conjecture that the Φ -order equation is the seminal model for photoreactions despite the fact that it has only been analytically derived for the primary photoprocess whose photoproduct is transparent to the radiation used (Maafi and Brown, 2007). Such a situation might be an analogue to that observed for thermal reactions where the mono-exponential function, obeyed by the kinetics of the simplest unimolecular reaction, is the basis for the description of kinetic data belonging to any other, more complex thermal reaction mechanisms. Incidentally, this is somewhat supported by the typical Φ -order equations set out for $C\left(t\right)$ by the semi-empirical method for several reactions (Maafi and Maafi, 2013; Maafi and Maafi, 2014a; Maafi and Maafi, 2014b; Maafi and Maafi, 2016).

The findings of the present work also allow us to put forward a conjecture stipulating that Φ -order kinetics (based on Eq. 4 template) maps out, in general, the behaviour of photoreaction kinetics (irrespective of the photomechanism considered, i.e., beyond Scheme 1).

Hence, the formulation of the integrated rate equation given by the proposed Eq. 4 becomes the unifying model in photokinetics. This represents the first time that a unique explicit description is proposed for the traces of photoreactions.

Conversely and even if some photokinetic traces may well be fitted by one or several mono-exponential functions, such an approach remains limited and cannot be generalised. It is unlikely to reach similar fitting results by using mono-exponential functions instead of Φ -order equations (of Eq. 4 template), keeping the kinetic meaning of the fitting results. It has already been shown that such an interchange of ( Φ -order/exponential) equations does not always work for the simplest primary photoreaction (Maafi and Brown, 2007). More importantly, this observation is fully justified, in principle, by the significant difference between the mathematical formulations of the rate laws describing photoreactions and thermal reactions. These differences in the differential equations lead to a predictable dissimilarity of the mathematical solutions that would be derived for each (e.g., first- and Φ -order kinetics).

In addition to the successful fitting of the traces of photoreactions by the unifying Eq. 4 model, this study has revealed an aspect that must be considered in any practical photokinetic investigation. Indeed, despite the constraints imposed on the fitting of the traces [as given by the coupling factor ${cc}^{\lambda_{irr}}$ and Eq. (7)], it has become evident that reaching a unique solution (a unique set of values for the fitting parameters ${cc}^{\lambda_{irr}}$ , $\omega_{ij}^{\lambda_{irr}}$ , and $k_{ij}^{\lambda_{irr}}$ ) for a given reaction is not achievable. This translates the occurrence of an identifiability issue (Vajda and Rabitz, 1988; Maafi and Brown, 2005; Hattersley et al., 2011; Ovchinnikov et al., 2023), i.e., changing the initial values where the fitting calculation starts, yields a quite large number of different sets of fitting values of the parameters. Each of these sets has equally excellent fitting metrics of the RK traces, indicating that the Levenberg–Marquardt algorithm can converge to different minima. The reason behind the identifiability issue is a smaller number of linearly independent equations describing the reactive system compared to the number of fitting parameters. In addition, Eq. (7) is linearly independent from Eq. 4, but is non-linear.

This point is well illustrated in the case of the simplest photoreaction ( $X\to Y_{1}$ , whose photoproduct is transparent to the irradiation light). Here, the trace is described by a mono- Φ -order equation. Nonetheless, it is not possible to single out the true solution of the system (defined by the true values of $\omega_{10}^{\lambda_{irr}}$ , ${cc}^{\lambda_{irr}}$ , and $k_{10}^{\lambda_{irr}}$ describing the reaction), where successive fittings (with different initial values) yield several possible sets of fitting parameters’ values with excellent fit of the trace. For more extended reactions, the insolvability of the identifiability issue becomes obviously more acute as the ratio between parameters’ and equations’ numbers becomes even larger. Overall, the fundamental problem posed here emerges from the unavailability of $\omega_{ij}^{\lambda_{irr}}$ , ${cc}^{\lambda_{irr}}$ , and $k_{ij}^{\lambda_{irr}}$ explicit expressions (which is itself due to the impossibility of an analytical integration of the system’s differential equations). This concept is easily verified for the case of the only reaction whose solution is analytically derived and the expressions of its parameters are known, namely, the primary photoprocess (Maafi and Brown, 2007). Indeed, among all possible sets of parameters of this reaction’s Eq. 4 (which is a mono- Φ -order equation model) that generate good fit of an RK trace, it is possible to single out the true solution set of fitting parameters’ values ( $\omega_{10}^{\lambda_{irr}}$ , ${cc}^{\lambda_{irr}}$ , and $k_{10}^{\lambda_{irr}}$ ) based on the fact that the equations of the latter coefficients are known (Maafi and Brown, 2007).

In this context, it is important to stress that such an identifiability issue cannot be solved by considering a combination of traces obtained at “any” number of different irradiation wavelengths, as might have been previously proposed (Crano and Guglielmetti, 2003; Delbaere et al., 2011; Micheau et al., 2014). The reason for this inconsistency is a proportional increase of the unknown parameters with each additional Eq. 4 when increasing the number of traces at different $\lambda_{irr}$ . The seemingly success of the aforementioned approach came at a cost: it must assume that the quantum yields of the individual reaction steps are necessarily invariants with $\lambda_{irr}$ (i.e., each species has a unique quantum yield for all $\lambda_{irr}$ , or $\Phi_{Y_{j}}^{\lambda_{irr}}=\Phi_{Y_{j}}$ ). The latter condition addresses a very particular situation, which, in any case, needs to be proven experimentally and not assumed (the description of the latter approach (Crano and Guglielmetti, 2003) has neither proven experimentally such an invariability of the quantum yield nor considered the necessity of recommending an experimental proof on the quantum yield invariability before envisaging its application).

In a previous study by our team (Maafi and Brown, 2008), detailed spectrokinetic methods have been proposed with the aim of solving the identifiability problem (i.e., leading to the true solutions) of photoreversible systems. They showed the limits of using several traces obtained for a reactive system exposed to different irradiation wavelengths.

For the temporal variation in the total absorbance of the reactive medium ( $A_{tot}^{\lambda_{irr}/\lambda_{obs}}\left(t\right)$ ) irradiated at a given $\lambda_{irr}$ and monitored at $\lambda_{obs}$ , where $\lambda_{irr}$ and $\lambda_{obs}$ may or may not be equal (i.e., $\varepsilon_{Y_{j}}^{\lambda_{obs}}$ and $l_{obs}$ might be different from those measured at $\lambda_{irr}$ ), Eq. 4 equivalent takes the following form:

$$A_{tot}^{\lambda_{irr}/\lambda_{obs}}\left(t\right)=A_{tot}^{\lambda_{irr}/\lambda_{obs}}\left(\infty \right)+\sum_{i=1}^{n_{\Phi A}}\omega_{i,A}^{\lambda_{irr}}\;\mathrm{Log}\left(1+{cc}_{A}^{\lambda_{irr}}e^{-k_{iA}^{\lambda_{irr}}\;t}\right).$$ (8)

Note that Eq. (8) has the template of Eq. 4 because the former is a linear combination of the latter for a given reactive system. Hence, the number of terms in Eq. (8) ( $i_{A}$ ) would, at most, be equal to the number of reaction steps occurring in the photoreaction ( $n_{\Phi }$ ). For the trace of $A_{tot}^{\lambda_{irr}/\lambda_{obs}}\left(t\right)$ (Eq. (8)), $A_{tot}^{\lambda_{irr}/\lambda_{obs}}\left(\infty \right)$ is the total absorbance of the reactive medium at the end of the reaction, the factor $\omega_{i,A}^{\lambda_{irr}}$ is equivalent to the $\omega_{ij}^{\lambda_{irr}}$ of the species individual traces in Eq. 4 multiplied by the respective constants $\varepsilon_{Y_{j}}^{\lambda_{obs}}\;l_{obs}$ , the factor ${cc}_{A}^{\lambda_{irr}}$ is also constant in all terms of Eq. (8), and the rate constants of the different reaction’s regimes, in Eqs. (4) and (8), are invariant, i.e., $k_{iA}^{\lambda_{irr}}=k_{ij}^{\lambda_{irr}}$ .

It is important, however, to mention that the total absorbance trace varies according to the conditions in which the measurement is performed. $A_{tot}^{\lambda_{irr}/\lambda_{obs}}$ is depending on which of the following combinations is considered: i) $\lambda_{irr}=\lambda_{obs}$ and $l_{irr}=l_{obs}$ ; ii) $\lambda_{irr}=\lambda_{obs}$ and $l_{irr}\neq l_{obs}$ ; iii) $\lambda_{irr}\neq \lambda_{obs}$ and $l_{irr}=l_{obs}$ ; iv) $\lambda_{irr}\neq \lambda_{obs}$ and $l_{irr}\neq l_{obs}$ . Hence, the properties of $A_{tot}^{\lambda_{irr}/\lambda_{obs}}\left(t\right)$ traces, and consequently, the fitting parameters, except $k_{i,A}^{\lambda_{irr}}$ , will change between situations (i–iv), for a given reactive system.

Overall, however, the fitting of the RK traces of given systems, with Eq. 4 models, will allow us to describe some general aspects of photokinetics under monochromatic light, as well as to construct a method to fully solve such a photokinetics even when $\Phi_{\lambda_{irr}}$ is variable with $\lambda_{irr}$ , as it will be discussed in the following sections.

### 3.6 Metrics in photokinetics

Basically, there are two types of coefficients that fully define the kinetics of a photoreaction: extrinsic and intrinsic coefficients. The former, such as $C_{X}\left(0\right)$ , $P_{0}^{\lambda_{irr}}$ , $l_{irr}$ , and $l_{obs}$ , are controlled by the experimentalist and represent the experimental conditions (assumed known). The latter ones are specific features of the different species involved in the photoreaction, i.e., $\varepsilon_{Y_{j}}^{\lambda_{irr}}$ , $\Phi_{Y_{j}\;\to \;Y_{j^{\prime }}}^{\lambda_{irr}}$ (with $j\neq j^{\prime }$ ).

In general, one of the metrics in kinetics is the reaction rate constants ( k ). However, this typical approach is compromised when the identifiability issue cannot be solved, as previously seen for the fitting of the photokinetic RK traces with Eq. 4. In order to circumvent such a hurdle, the use of the initial reaction rate of the reactant and/or those of the species emanating from the reactant would represent a more appropriate means to quantifying the variability of the reaction speed.

It is to be noted that this metric (e.g., $RK:r_{0X}^{\lambda_{irr}}$ ) is the only accessible trace parameter by numerical integration (the rate constants of the reaction regimes are, in general, not calculated by NIMs and not possibly calculated by the experimentalist from those traces or NIM-fed parameters since their explicit formulae are not known). Therefore, since the initial species’ rates can readily be calculated directly by NIMs ( $RK:r_{0}^{\lambda_{irr}}$ ) for all reactive species, they become good photokinetic metric tools, indicative of the reaction performance. The proportionality between $r_{0X}^{\lambda_{irr}}$ and $k_{iX}^{\lambda_{irr}}$ , as indicated by Eq. (6), validates the characterisation of the reactant’s photoreactivity by its initial rate.

Overall, the speed up (or the slowdown) of the reactant photoconversion is expected to be followed by the rest of the reaction species’ reactivities in relative proportions (for instance, a change in the rate when a value of an extrinsic coefficient is changed). Consequently, the variation in the reactant speed in one direction is indicative of the variation in the velocities of each of the subsequent species of the reaction in the same direction.

For the present study, another way is offered for the determination of the initial reaction rate, that is, by applying Eq. 4 to the RK trace of the reactant. The negative initial reactant rate [Eq. (9)], for the Φ -shaped reaction mechanism (Scheme 1) where X is depleted to form $Y_{1}$ and $Y_{3}$ , can be worked out from the following expression:

$$Fit:r_{0X}^{\lambda_{irr}}=Fit:r_{0Y_{1}}^{\lambda_{irr}}+Fit:r_{0Y_{3}}^{\lambda_{irr}}=-\frac{1}{\ln\left(10\right)}\;\left(\frac{{cc}^{\lambda_{irr}}}{1+{cc}^{\lambda_{irr}}}\right)\sum_{i=1}^{i_{0}}\omega_{i0}^{\lambda_{irr}}\;k_{i0}^{\lambda_{irr}},$$ (9)

where the individual, positive, and initial photoproduct rates ( $Fit:r_{0Y_{1}}^{\lambda_{irr}}$ and $Fit:r_{0Y_{3}}^{\lambda_{irr}}$ ) are obtained from their respective Eq. 4 as

$$Fit:r_{0Y_{1}}^{\lambda_{irr}}=-\frac{1}{\ln\left(10\right)}\;\left(\frac{{cc}^{\lambda_{irr}}}{1+{cc}^{\lambda_{irr}}}\right)\;\sum_{i=1}^{i_{1}}\omega_{i1}^{\lambda_{irr}}\;k_{i1}^{\lambda_{irr}},$$ (10)

$$Fit:r_{0Y_{3}}^{\lambda_{irr}}=-\frac{1}{\ln\left(10\right)}\;\left(\frac{{cc}^{\lambda_{irr}}}{1+{cc}^{\lambda_{irr}}}\right)\;\sum_{i=1}^{i_{3}}\omega_{i3}^{\lambda_{irr}}\;k_{i3}^{\lambda_{irr}}.$$ (11)

It is important to notice that the values of $Fit:r_{0}^{\lambda_{irr}}$ obtained in this way will not vary for the different sets of fitting parameters possibly generated for the RK traces (due to the occurrence of the identifiability issue), as long as each of the sets produces an excellent fit of the RK traces by the corresponding Eq. 4.

An additional way to confirm the validity of the aforementioned equations is to compare the values they generate with those obtained from the theoretical rate law of the reaction [Eq. (1)]. The values of $Theo:r_{0X}^{\lambda_{irr}}$ , $Theo:r_{0Y_{1}}^{\lambda_{irr}}$ , and $Theo:r_{0Y_{3}}^{\lambda_{irr}}$ can be obtained from the following equation:

$$r_{0X}^{\lambda_{irr}}=-r_{0Y_{1}}^{\lambda_{irr}}-r_{0Y_{3}}^{\lambda_{irr}}=-\left(\Phi_{X\to Y_{1}}^{\lambda_{irr}}+\Phi_{X\to Y_{3}}^{\lambda_{irr}}\right)\;P_{0}^{\lambda_{irr}}\;\left(1-10^{-A_{X}^{\lambda_{irr}}\left(0\right)}\right).$$ (12)

**FIGURE 2:**
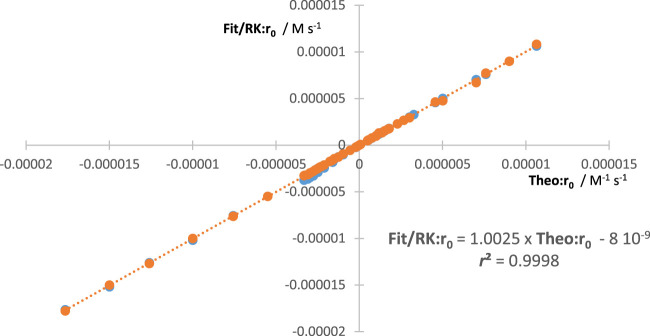
Linear correlation between the values of $Theo:r_{0Y_{j}}^{\lambda_{irr}}$ against both $RK:r_{0Y_{j}}^{\lambda_{irr}}$ and $Fit:r_{0Y_{j}}^{\lambda_{irr}}$ . Data belong to more than 60 reaction cases.

The reliability of our methodology should be confirmed by the equality $Fit:r_{0Y_{j}}^{\lambda_{irr}}=Theo:r_{0Y_{j}}^{\lambda_{irr}}=RK:r_{0Y_{j}}^{\lambda_{irr}}$ in all circumstances (Figure 2).

Accordingly, and despite the identifiability problem, it is possible to consistently evaluate the kinetics of a photoreaction, and therefore, $r_{0}^{\lambda_{irr}}$ becomes one of the essential metric tools of photokinetics. In the case of a total absorbance trace, the initial velocity can be obtained from the following expression:

$$\begin{array}{c} Fit:r_{0A}^{\lambda_{irr}}=-\frac{1}{\ln\left(10\right)}\;\left(\frac{{cc}_{A}^{\lambda_{irr}}}{1+{cc}_{A}^{\lambda_{irr}}}\right)\;\sum_{i=1}^{i_{A}}\omega_{iA}^{\lambda_{irr}}\;k_{iA}^{\lambda_{irr}}\\ =Theo:r_{0A}^{\lambda_{irr}}=l_{irr\;or\;obs}\sum_{j=0}^{1,3}\varepsilon_{Y_{j}}^{\lambda_{irr\;or\;obs}}\;r_{0Y_{j}}^{\lambda_{irr}}. \end{array}$$ (13)

It is to be noted that the expressions for the initial rates of the reactant and photoproducts ( X , $Y_{1}$ , and $Y_{3}$ ) cannot be obtained from the fitting of the total absorbance trace [second term of Eq. (13)] because the theoretical equation of the initial rate is a combination of terms as given by the last term of Eq. (13).

Nonetheless, $r_{0A}^{\lambda_{irr}}$ is an as good metric for photokinetics as was $Fit:r_{0X}^{\lambda_{irr}}$ , but with a considerable advantage, that is, of relieving the experimentalist from obtaining the individual species $C_{Y_{j}}^{\lambda_{irr}}\left(t\right)$ traces ( $A_{tot}^{\lambda_{irr}/\lambda_{obs}}\left(t\right)$ can be generated on a routine spectrophotometer, preferably with an *in situ* irradiation).

### 3.7 Contribution of spectators to photo-rates

A spectator molecule (one of w molecules, SPM_*r*_, with w≥r≥1 ) is a species present in the reactive medium but does not interact with reactants and products of the photoreaction, has a constant concentration, is photo- and thermally inert, but absorbs at the irradiation wavelength. In thermal kinetics, a spectator of this type would have no effect on the reactivity of the species involved in the reaction mechanism. However, it is different in photokinetics. In the cases where a non-zero absorbance of the spectator molecule(s) at $\lambda_{irr}$ , $A_{{SPM}_{r}}^{\lambda_{irr}}\neq 0$ (Eq. (14)), is considered, it must necessarily make part of the total absorbance of the medium (Eqs. 3, 14), since the fraction of the light absorbed by the spectators is lost for the reactive species. It is to be noted that no conditions on $A_{{SPM}_{r}}^{\lambda_{irr}}$ to belong to the linearity ranges of the ${SPM}_{r}$ calibration graph are necessary, since $A_{{SPM}_{r}}^{\lambda_{irr}}$ does not contribute to the mathematical formalism of the photokinetics other than by its actual constant value.

$$A_{tot}^{\lambda_{irr}}\left(t\right)=\sum_{r=1}^{w}A_{{SPM}_{r}}^{\lambda_{irr}}+\sum_{j=0}^{n_{sp}}A_{Y_{j}}^{\lambda_{irr}}\left(t\right).$$ (14)

**FIGURE 3:**
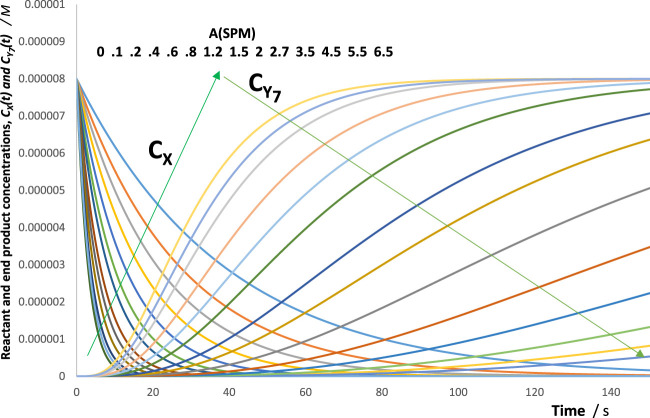
Evidence of a gradual decrease in X and $Y_{7}$ reactions’ rates upon an increase of the spectator molecules’ absorbance at the irradiation wavelength, $A_{{SPM}_{r}}^{\lambda_{irr}}$ . The values of $A_{{SPM}_{r}}^{\lambda_{irr}}$ are shown. The arrows indicate the directions of evolution of each species traces with increasing values of $A_{{SPM}_{r}}^{\lambda_{irr}}$ . The data correspond to a multi-consecutive reaction involving four photoproducts, as shown in Figure 4.

**FIGURE 4:**
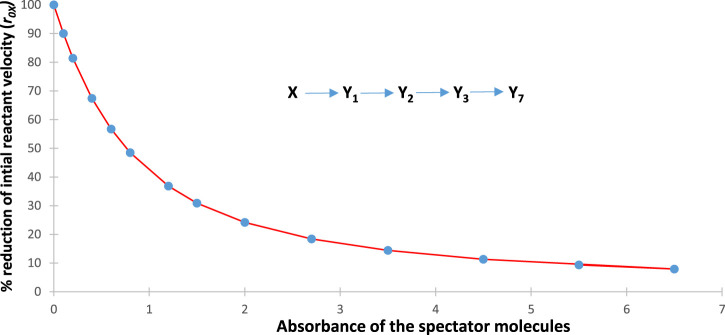
Sequential percentage reduction of the reactant initial speed with an increase in the absorbance of the spectator molecules ( $A_{{SPM}_{r}}^{\lambda_{irr}}$ ) present in the reactive medium. The percentage reduction of $r_{0X}^{\lambda_{irr}}$ , represented by the dots, was obtained by using RK-calculated and Eq. 4 ( $RK:r_{0X}^{\lambda_{irr}}$ and $Fit:r_{0X}^{\lambda_{irr}}$ ) initial reactant-rate values, and the line corresponds to $Theo:r_{0X}^{\lambda_{irr}}$ given by Eq. 12.

For this reason, an increase in the absorbance of the spectators is expected to result in a decrease of all species’ reactivities (i.e., rate constants and initial rates). Figure 3 depicts such a behaviour, where the rate of the reactant X and photoproduct, $Y_{7}$ , are reduced with an increase in the concentration of the spectator for the illustration mechanism shown in Figure 4 (a similar trend is also observed for the rest of the species rates, whose traces are not shown in Figure 3).

This expectation has turned out to be true for all the cases investigated. It corroborates the experimental results obtained for the primary photoprocess ( $X\to \;Y,\varepsilon_{Y}=0$ ) (Maafi and Maafi, 2013; Maafi and Maafi, 2015a) and the photoreversible reaction (Maafi and Maafi, 2015b), where the effects were quantitatively demonstrated by plotting the rate constant *versus* the photokinetic factor. However, in the present study, the evidence is provided by a progressive decrease in the initial reactant rate with an increase in SPM concentration.

As shown in Figure 4, each data point corresponds to both $RK:r_{0X}^{\lambda_{irr}}$ and $Fit:r_{0X}^{\lambda_{irr}}$ , whereas the curve joining the data points corresponds to $Theo:r_{0X}^{\lambda_{irr}}$ as functions of $A_{{SPM}_{r}}^{\lambda_{irr}}$ .

Beyond an established effect of the spectator on photoreactivity, it is important to stress a practical aspect that might be found useful for some applications. In other words, the photoreactivity of a molecule can effectively and significantly be reduced or even virtually stopped by an adequate selection of the concentration of one or more convenient spectator molecules. The reduction in photoreactivity by the presence of a spectator molecule has widely been exploited in chromatic orthogonality by performing a particular reaction within multi-component mixtures, though this use of SPM was treated only through a qualitative approach that has not been mathematically formalised (Bochet, 2006; Hansen et al., 2015).

### 3.8 $C_{X}^{\lambda_{irr}}\left(0\right)$ , $l_{irr}$ , and $\varepsilon_{X}^{\lambda_{irr}}$ effects on photoreactivity

The photokinetics of reactions must be dependent on the initial concentration of the starting reactant owing to the rate law [Eq. (1)], and more specifically, its photokinetic factor [Eq. (2)], being a function of concentration. Theoretically, the Φ -order kinetic pattern of the traces should be preserved when changing $C_{X}^{\lambda_{irr}}\left(0\right)$ , but the rate of the reaction should be affected by the value of $C_{X}^{\lambda_{irr}}\left(0\right)$ (all remaining extrinsic factors being the same), together with the initial reactant rate (as our metric tool in this work). It is also predictable that such an effect of $C_{X}^{\lambda_{irr}}\left(0\right)$ on the rate of the reactant would be passed on to the photokinetics of the photoproducts whose $r_{ij}^{\lambda_{irr}}$ would be proportionally changed according to their particular kinetic properties (since their rate laws depend on the concentrations of all species including that of X ). In this configuration, an increase in $-r_{0_{X}}^{\lambda_{irr}}$ is expected for an increased $C_{X}^{\lambda_{irr}}\left(0\right)$ (i.e., a higher $C_{X}^{\lambda_{irr}}\left(0\right)$ leads to a higher $A_{tot}^{\lambda_{irr}}\left(0\right)=A_{X}^{\lambda_{irr}}\left(0\right)$ and, hence, higher values of [ $1-10^{-A_{X}^{\lambda_{irr}}\left(0\right)}$ ), Eq. (1)]. At the same time, increasing $C_{X}^{\lambda_{irr}}\left(0\right)$ will cause a reduction in the rate constants ( $k_{ij}^{\lambda_{irr}}$ ) due to smaller values of PKF (Eq. (2), when $A_{tot}^{\lambda_{irr}}\left(t\right)$ increases) as was suggested by the semi-empirical method (Maafi and Maafi, 2013; Maafi and Maafi, 2014a; Maafi and Maafi, 2014b; Maafi and Maafi, 2015a).

**FIGURE 5:**
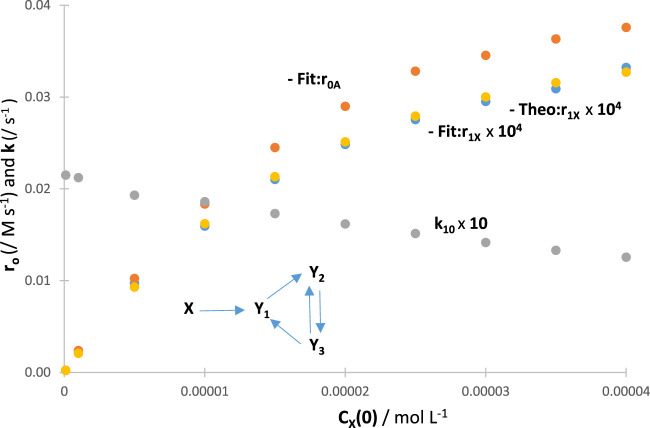
Variation in the initial reactant rates $RK:r_{0X}^{\lambda_{irr}}$ and $Fit:r_{0X}^{\lambda_{irr}}$ , the initial rate of the total absorbance $Fit:r_{0A}^{\lambda_{irr}}$ , and the fitting rate constant ( $k_{10}^{\lambda_{irr}}$ ) of the traces, with an increase in reactant initial concentration, for the indicated reaction.

A confirmation of $C_{X}^{\lambda_{irr}}\left(0\right)$ effects on $r_{0X}^{\lambda_{irr}}$ was investigated by performing RK calculations. The absolute values of $RK:r_{0X}^{\lambda_{irr}}$ for a given reaction increase with an increase in the values of $C_{X}^{\lambda_{irr}}\left(0\right)$ , tending asymptotically towards a limit (the latter should be reached at a total absorption of the light). When the RK traces were fitted by the appropriate Eqs 4 or 8, a gradual reduction in $k_{ij}^{\lambda_{irr}}$ values was recorded with an increase in the values of $C_{X}^{\lambda_{irr}}\left(0\right)$ (Figure 5). These trends were independent of the actual mechanism of the photoreaction (an example is provided in Figure 5). Such general results on the effect of $C_{X}^{\lambda_{irr}}\left(0\right)$ set out a fundamental photokinetic property, which does not seem to have been reported, thus far, in the literature as a general feature, i.e., irrespective of the mechanism of the reaction at hand. The auto-photostabilisation of the reaction with increasing $C_{X}^{\lambda_{irr}}\left(0\right)$ has previously been experimentally evidenced for two reactions (using semi-empirical equations), namely, the primary photoprocess with an $\varepsilon_{Y_{1}}^{\lambda_{irr}}\neq 0$ (Maafi and Maafi, 2013; Maafi and Maafi, 2014a) and the photoreversible reaction (Maafi and Maafi, 2015a), whose $k_{10}^{\lambda_{irr}}$ linearly correlated with $PKF\left(0\right)$ . Incidentally, it has been observed that the ratios of pairs of species’ final concentrations (end products or photostationary species) were independent of the values of $C_{X}^{\lambda_{irr}}\left(0\right)$ .

The results obtained for $RK:r_{0X}^{\lambda_{irr}}$ have served to the endorsement of both $Fit:r_{0X}^{\lambda_{irr}}$ and $Theo:r_{0X}^{\lambda_{irr}}$ results (Figure 5), hence confirming the usefulness and reliability of the proposed model equation (Eq. 4).

Furthermore, since $l_{irr}$ is imbedded within the absorption formula [always multiplying the concentration in Eq. (1)], its variation will cause similar photokinetic effects to those described previously for a change in $C_{X}^{\lambda_{irr}}\left(0\right)$ . This was observed by monitoring $RK:r_{0X}^{\lambda_{irr}}$ and $Fit:r_{0X}^{\lambda_{irr}}$ , indicating that Eq. 4 applies irrespective of the reactor’s length.

It is to be noted that the effect of varying $C_{X}^{\lambda_{irr}}\left(0\right)$ and/or $l_{irr}$ is equivalent to when a similar change in the $\varepsilon^{\lambda_{irr}}$ values occurs, since the former quantities always multiply the latter in Eq. (1) (within the expressions of absorbances). Therefore, investigating a change in $C_{X}^{\lambda_{irr}}\left(0\right)$ both informs about the trend of varying $l_{irr}$ and equates to studying a class of a reactive system, whose reactant and photoproduct absorption coefficients ( $\varepsilon^{\lambda_{irr}}$ ) were proportionally changed. This might be of interest when designing a photoreactive system for particular applications.

### 3.9 Photokinetic impact of $P_{0}^{\lambda_{irr}}$ , $S_{irr}$ , and $V_{irr}$ : kinactinometry

For the purpose of describing how the radiation intensity within the reactor, $P_{0}^{\lambda_{irr}}$ , might affect the photoreaction reactivity, it is useful to define what this quantity stands for.

As $P_{0}^{\lambda_{irr}}$ is certainly related to the energy supplied by the light source, the latter is a good starting point.

The incident radiation of the monochromatic light beam, which can be measured by a physical actinometer as a spectral irradiance of the light source, $E_{sp-irr}$ , is expressed in $W\;cm^{-2}\;nm^{-1}$ or $J\;s^{-1}\;cm^{-2}\;nm^{-1}$ (i.e., energy ( J ) per unit time, t ( s ), per unit area [1 $cm^{2}$ ), and per wavelength, $\lambda_{irr}$ ( nm )]. Since the radiation must be monochromatic for the kinetic model considered in the present work, the measured spectral irradiation corresponds to a single wavelength. This allows to ignore the unit, per wavelength ( $nm^{-1}$ ), in the dimension of the spectral irradiation. The unit J/s corresponds to an energy flux.

In experimental photochemistry, the number of photons is preferred to the energy of the beam since the former allows an adequate quantitative metric because the reaction occurs between this number of photons and the number of photoactive species in the medium. This number of photons is determined as follows.

The energy ( $E_{h\nu }^{\lambda_{irr}}$ in J ) carried by one photon of the radiation is a function of the wavelength $\lambda_{irr}$ .

$$E_{h\nu }^{\lambda_{irr}}=\frac{h\;c}{\lambda_{irr}},$$ (15)

where h is Planck’s constant expressed in J s ( $h=6.62608\;10^{-34}\;J\;s$ ), c is the velocity of light given in m/s ( $299792458\;m\;s^{-1}$ ), and $\lambda_{irr}$ is the wavelength in m .

For an einstein (a mole) of photons of a given wavelength (or Avogadro’s number of photons per mole, $N_{a}$ , $N_{a}=6.02214\;10^{23}$ , in $einstein^{-1}$ ), carrying a total energy $E_{mol\;h\nu }^{\lambda_{irr}}$ , the latter equation becomes

$$E_{mol\;h\nu }^{\lambda_{irr}}=N_{a}\frac{h\;c}{\lambda_{irr}}.$$ (16)

Applying this formula to the physically measured energy of the radiation, provided by the spectral irradiance, $E_{sp-irr}$ , gives the number of photons (number of einstein or moles of photons) carried by the radiation per units of time and area. This quantity represents the incident photon flux of the monochromatic light source through a unit area, ${flx}_{h\nu }^{\lambda_{irr}}$ .

$${flx}_{h\nu }^{\lambda_{irr}}=\frac{E_{sp-irr}}{E_{mol\;h\nu }^{\lambda_{irr}}}=\frac{E_{sp-irr}\;\lambda_{irr}}{N_{a}h\;c}.$$ (17)

The dimension analysis of Eq. (17), $\left[{flx}_{h\nu }^{\lambda_{irr}}\right]=\left[E_{sp-irr}\;\lambda_{irr}/N_{a}h\;c\right]=\left(J\;s^{-1}\;{cm}^{-2}\;m\right)/\left(einstein^{-1}\;J\;s\;m\;s^{-1}\right)=einstein\;s^{-1}\;{cm}^{-2}$ , confirms a flux dimension ( $einstein\;s^{-1}$ ) per unit area.

Therefore, in order to express the number of photons entering the reactor per s for a given experimental setup, with the specific irradiated area of the sample, it is necessary to multiply the incident flux per unit surface $\left({flx}_{h\nu }^{\lambda_{irr}}\right)$ , by the actual irradiated area of the sample, $S_{irr}$ (in ${cm}^{2}$ ).

$${flx}_{h\nu ,S_{irr}}^{\lambda_{irr}}={flx}_{h\nu }^{\lambda_{irr}}\;S_{irr}.$$ (18)

Finally, ${flx}_{h\nu ,S_{irr}}^{\lambda_{irr}}$ is to be reported to the actual volume ( $V_{irr}$ in ${dm}^{3}$ ) of the sample subjected to irradiation. The derived quantity, representing the $P_{0}^{\lambda_{irr}}$ in Eq. (2), is expressed as

$$P_{0}^{\lambda_{irr}}=\frac{{flx}_{h\nu }^{\lambda_{irr}}\;S_{irr}}{V_{irr}},$$ (19)

whose dimension analysis gives $\left[P_{0}^{\lambda_{irr}}\right]=\left(einstein\;s^{-1}\;{cm}^{-2}\;{cm}^{2}\right)/\left({dm}^{3}\right)=einstein\;s^{-1}\;{dm}^{-3}$ . $P_{0}^{\lambda_{irr}}$ is the number of photons entering the reactor per second, per irradiated area and volume of the investigated sample.

The dimension of $P_{0}^{\lambda_{irr}}$ is adequate for a rate law describing a photochemical reaction [Eq. (1)]. The dimension characterising the left-hand side term of that differential equation, corresponding to a differentiation of the concentration with time, as $mol/\left(L\;s\right)$ or $mol\;{dm}^{-3}\;s^{-1}$ , and the right-hand term of the equation has the unit of $P_{0}^{\lambda_{irr}}$ (both quantum yields, $\Phi^{\lambda_{irr}}$ , and factors multiplying $P_{0}^{\lambda_{irr}}$ [Eq. (2)] are dimensionless). Therefore, the same unit is found for either side of the differential equation (the similarity claim is consistent, since for photons count, mol is equivalent to einstein ).

The IUPAC (Braslavsky, 2007) defines a quantity $q_{n,p,\lambda }^{0}$ as the radiation spectral photon flux on amount basis, with a dimension of $einstein\;s^{-1}\;{nm}^{-1}$ or $einstein\;s^{-1}$ for a monochromatic light. In the expression given therein for the rate law, the spectral photon flux is divided by the volume irradiated ( $q_{n,p,\lambda }^{0}/V$ , in $einstein\;s^{-1}\;{dm}^{-3}$ ). The latter quantity has the same dimension as $P_{0}^{\lambda_{irr}}$ [Eq. (19)]. However, it is to be noted that Eq. (19) takes into account the actual irradiated area of the sample (the two equations will be equal if $S_{irr}=1\;{cm}^{2}$ ).

Therefore, the value of $P_{0}^{\lambda_{irr}}$ can change due to a variation in either the incident flux of the light source or lamp ( ${flx}_{h\nu }^{\lambda_{irr}}$ ), the irradiated area ( $S_{irr}$ ), or the volume ( $V_{irr}$ ) of the sample exposed to the light. In this context, it is obviously important to ensure a vigorous stirring of the reactive medium, throughout irradiation, for consistency with the value of $V_{irr}$ in Eq. (19). It is to be noted also that the ratio $\left(V_{irr}/S_{irr}\right)$ , in Eq. (19), is not necessarily equal to the optical path length of the light beam inside the sample $l_{irr}$ (it would only be the case if the whole volume is exposed to the light through one of its full sides, but in general, $V_{irr}/S_{irr}\geq l_{irr}$ ).

This, then, commends that the three properties are reported in the investigation in order to complete the adequate photokinetic analysis. Unfortunately, in many published studies, these quantities are missing. This might explain, at least in part, the difficulties in comparing the photokinetic results that were obtained in different laboratories.

Practically, the changes in ${flx}_{h\nu }^{\lambda_{irr}}$ , $S_{irr}$ , and $V_{irr}$ can be controlled by the experimentalist by, respectively, varying the electric potential of the lamp, the blinded surface, or volume irradiated of the sample (these are part of the extrinsic parameters).

The qualitative effect of increasing $P_{0}^{\lambda_{irr}}$ is an increase of the photoreaction reactivity. The quantitative assessment of such an effect is provided by proportional changes in the values of $k_{ij}^{\lambda_{irr}}$ (as proven in the previous work (Maafi and Maafi, 2013; Maafi and Maafi, 2014a; Maafi and Maafi, 2014b; Maafi and Lee, 2015a; Maafi and Maafi, 2015a; Maafi and Lee, 2015b; Maafi and Maafi, 2016; Maafi and Al-Qarni, 2019)). In addition, an increase in $P_{0}^{\lambda_{irr}}$ leads to a reduction in the overall time necessary for the reaction to reach completion and, hence, a reduction in $t_{1/2}$ . Furthermore, a variation in $P_{0}^{\lambda_{irr}}$ causes a proportional change in the value of $r_{0}^{\lambda_{irr}}$ .

The determination of the values of $P_{0}^{\lambda_{irr}}$ is essential for quantitative photochemistry and fundamental in converting a reactive system into an actinometer. The value of $P_{0}^{\lambda_{irr}}$ is obtained from Eq. (20) [which is a rearranged Eq. 12]. Hence, photokinetic data are used to perform actinometry, and this methodology is dubbed here: kinactinometry.

$$P_{0}^{\lambda_{irr}}=\frac{-Theo:r_{0X}^{\lambda_{irr}}\;\left(or\;Fit:r_{0X}^{\lambda_{irr}}\right)}{\left(\Phi_{X\to Y_{1}}^{\lambda_{irr}}+\Phi_{X\to Y_{3}}^{\lambda_{irr}}\right)\;\left(1-10^{-A_{X}^{\lambda_{irr}}\left(0\right)}\right)}.$$ (20)

Actinometry is the measure of the incident light flux per unit area and volume ( $P_{0}^{\lambda_{irr}}$ ) at $\lambda_{irr}$ , entering a given reactor (a slab-shaped reactor for our study), and a chemical actinometer is a standardised reactive system that can deliver $P_{0}^{\lambda_{irr}}$ . The standardisation can be achieved kinetically by quantifying the linearly correlated variation of $P_{0}^{\lambda_{irr}}$ with the rate constant of a given reaction step ( $k_{ij}^{\lambda_{irr}}$ ) of the global photoreaction, and/or the initial reactant rate ( $r_{0X}^{\lambda_{irr}}$ or $r_{0A}^{\lambda_{irr}}$ , the metric in this study). Such a linear correlation is an advantage provided by a description of the photokinetic traces with equations of the type of Eq. 4. The correlation between the number of photons entering the reactor, $P_{0}^{\lambda_{irr}}$ , and $r_{0Y_{j}}^{\lambda_{irr}}$ follows from the relationship between $P_{0}^{\lambda_{irr}}$ and the number of photons absorbed by the considered species, $P_{aY_{j}}^{\lambda_{irr}}$ .

Technically, when varying $P_{0}^{\lambda_{irr}}$ , the model equations (Eq. 4) of the different species of the reaction share the same factors $C_{\infty ,j}^{\lambda_{irr}}$ , $\omega_{ij}^{\lambda_{irr}}$ , and ${cc}^{\lambda_{irr}}$ since all are independent of $P_{0}^{\lambda_{irr}}$ . The final concentrations (at t=∞ ) of the reactive species ( $C_{\infty ,j}^{\lambda_{irr}}$ ) are invariant with $P_{0}^{\lambda_{irr}}$ , but they are reached faster (shorter $t_{1/2}$ ) for higher values of $P_{0}^{\lambda_{irr}}$ . Hence, changing $P_{0}^{\lambda_{irr}}$ will induce a proportional change in the $k_{ij}^{\lambda_{irr}}$ factors, as the only changing fitting parameter, which adds robustness of the methodology and, overall, eases the fitting process.

**FIGURE 6:**
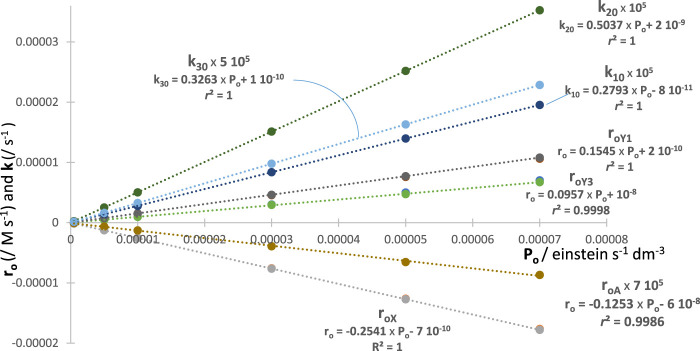
Linear correlations of the $k_{i0}^{\lambda_{irr}}$ and $r_{0,X}^{\lambda_{irr}}$ , $r_{0,Y_{1}}^{\lambda_{irr}}$ , $r_{0,Y_{3}}^{\lambda_{irr}}$ , and $Fit:r_{0,A}^{\lambda_{irr}}$ with various $P_{0}^{\lambda_{irr}}$ values for a reaction governed by the Φ -shaped mechanism shown in Scheme 1. Each point of $r_{0,Y_{j}}^{\lambda_{irr}}$ in the plot cumulates RK, Theo, and Fit values (fitting $C_{X}^{\lambda_{irr}}\left(t\right)$ , $C_{Y_{1}}^{\lambda_{irr}}\left(t\right)$ and $C_{Y_{3}}^{\lambda_{irr}}\left(t\right)$ traces). The $k_{i0}^{\lambda_{irr}}$ factors were obtained from Eq. 4 corresponding to separate fittings of the individual $C_{X}^{\lambda_{irr}}\left(t\right)$ traces. The $r_{0,Y_{j}}^{\lambda_{irr}}$ factors were calculated from the differentiated species’ equations at t=0 (Eqs 9–13).

Since the aforementioned behaviour is expected for each of $r_{0Y_{j}}^{\lambda_{irr}}$ factors (e.g., for X , $Y_{1}$ , and $Y_{3}$ ), then each is a good metric tool for actinometry (with each $r_{0Y_{0,1\;or\;3}}^{\lambda_{irr}}$ factor having a different proportionality to $P_{0}^{\lambda_{irr}}$ ). A feature that can be beneficial experimentally as it means that a single species’ trace (e.g., the reactant’s) would be sufficient for achieving an actinometric measurement. In addition, it is important to use the total absorbance trace to demonstrate its usefulness for actinometry. It turns out that the fitting parameters, $k_{ij}^{\lambda_{irr}}$ factors and $r_{0A}^{\lambda_{irr}}$ , of the total absorbance traces with increasing $P_{0}^{\lambda_{irr}}$ values also deliver linear correlations (Figure 6). This is an additional confirmation of the validity of the model, but, as importantly, it proves the usefulness of a simplified way to experimentally achieve actinometry. Indeed, all is required is to fit the $A_{tot}^{\lambda_{irr}}\left(t\right)$ trace with Eq. 4. This kinactinometric approach has never been reported in the literature.

The excellent linear correlation of $P_{0}^{\lambda_{irr}}$ with $RK:r_{0X}^{\lambda_{irr}}$ and $Theo:r_{0X}^{\lambda_{irr}}$ stands for a confirmation of that proportionality based on RK calculations. The similar correspondence found with $Fit:r_{0X}^{\lambda_{irr}}$ and $Fit:r_{0A}^{\lambda_{irr}}$ is not only another validation of the proposed Eq. 4 but also a simple means to perform actinometry and recruit new actinometers. For a given experiment, the value $P_{0}^{\lambda_{irr}}$ is worked out from the specific equation (e.g., examples given in Figure 6) corresponding to the quantity measured from the fitting equation.

Experimentally, one needs to determine the value of $P_{0}^{\lambda_{irr}}$ . Reference and precise values of $P_{0}^{\lambda_{irr}}$ , at given wavelengths, can also be determined by using the set of reliable actinometers that were previously proposed by our team on the basis of semi-empirical equations (i.e., Eq. 4 type (Maafi and Lee, 2015a; Maafi and Maafi, 2015b; Maafi and Maafi, 2013; Maafi and Maafi, 2014a; Maafi and Maafi, 2014b, Maafi and Maafi, 2015a, Maafi and Maafi, 2016; Maafi and Al-Qarni,2019)).

**FIGURE 7:**
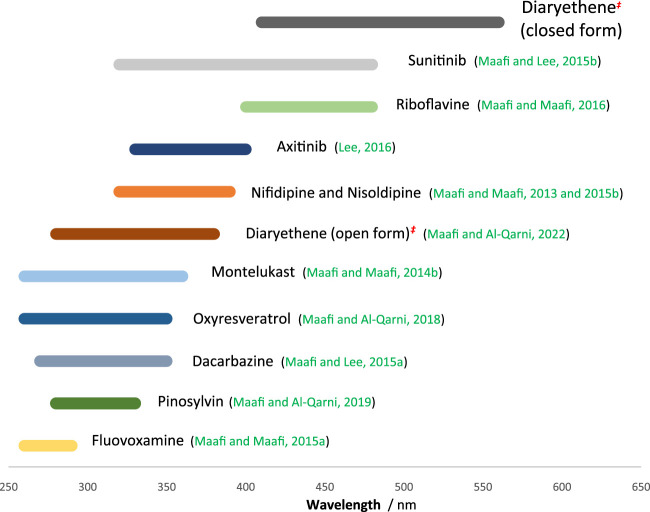
Spectral ranges for a series of existing efficient actinometers.

The large spectral region coverage by these actinometers (ranging between 250 and 580 nm, Figure 7) is useful for virtually any organic photoactive molecules. Because the implementation of these actinometers is very easy, requiring straightforward kinactinometric methods, they are strong alternatives to the ferrioxalate actinometer whose experimental setup is much more demanding. The list of these actinometers (Figure 7) can be extended by new (cheaper, water soluble, nanocarrier, etc.) candidates using the kinactinometric method proposed in the present work.

### 3.10 On the reactant’s quantum yield determination

The quantum yield of a reaction step ( $\Phi_{Y_{j}\to Y_{j^{\prime }}}^{\lambda_{irr}}$ ) is an intrinsic feature of the reagent $Y_{j}$ for the reaction step $Y_{j}\to \;Y_{j^{\prime }}$ at the irradiation wavelength considered. The monochromatic light is a requirement for the determination of the absolute values of the quantum yield (Braslavsky, 2007). Traditionally, $\Phi_{Y_{j}\to Y_{j^{\prime }}}^{\lambda_{irr}}$ was held as the most important characteristic of a photoreaction (Crano and Guglielmetti, 2003; Tonnesen, 2004; Pianowski, 2022). However, photokinetic analyses have shown that $\Phi_{Y_{j}\to Y_{j^{\prime }}}^{\lambda_{irr}}$ is a very important factor in defining the reactivity of a photospecies, nonetheless, in only a partial capacity, as it is one of a set of other elements [Eq. (1)]. The explicit expression of the primary photoprocess rate constant, which has been analytically derived (Maafi and Brown, 2007), indicates that the photochemical quantum yield is, at least, one of the four factors defining the reaction rate constant ( $k_{X}^{\lambda_{irr}}=\Phi_{X}^{\lambda_{irr}}\;\varepsilon_{X}^{\lambda_{irr}}\;P_{0}^{\lambda_{irr}}\;l_{irr}\;\ln$ 10). A similar conclusion has been reached from the expressions of rate constants of the unimolecular (Maafi and Maafi, 2013), reversible (Maafi and Maafi, 2014a), and multiconsecutive (Maafi and Maafi, 2016) photoreactions (where the $k_{ij}^{\lambda_{irr}}$ formulae have been obtained by a semi-empirical method).

In experimental photochemistry, several methods have been proposed for the determination of the quantum yield, albeit commonly based on actinometric measurements combined with the total absorption of the light by the reactive medium. A few examples of photokinetically determined absolute $\Phi_{Y_{j}\to Y_{j^{\prime }}}^{\lambda_{irr}}$ values have been earlier reported (Crano and Guglielmetti, 2003; Tonnesen, 2004; Pianowski, 2022). The advantage of the photokinetic–actinometric method (or kinactinometric method) is its applicability to relatively low concentrated actinometric solutions (as imposed by the linearity range of the calibration graph, which also means partial absorption of light by the medium) and its ability for the determination of a quantum yield value for each of the individual reaction steps occurring in the overall mechanism (Maafi and Lee, 2015a; Maafi and Lee, 2015b; Maafi and Maafi, 2013; Maafi and Maafi, 2014a; Maafi and Maafi, 2014b; Maafi and Maafi, 2015a; Maafi and Maafi, 2016; Maafi and Al-Qarni,2019).

Achieving such results (determination of $\Phi_{Y_{j}\to Y_{j^{\prime }}}^{\lambda_{irr}}$ from the analysis of the traces) through the usage of Eq. 4 might seem, at this stage, impossible because the explicit general formula of the factors $k_{ij}^{\lambda_{irr}}$ is unknown. Alternatively, in principle, it would be possible to develop a semi-empirical method for each mechanism in order to work out the absolute values of the different $\Phi_{Y_{j}\to Y_{j^{\prime }}}^{\lambda_{irr}}$ , but this approach would be time consuming.

It is, however, possible to exploit the model Eq. 4 for the determination of the reactant quantum yield. This is achievable based on Eqs. (21) and (22) (assuming that $P_{0}^{\lambda_{irr}}$ is known).

$$\Phi_{X\to Y_{1}}^{\lambda_{irr}}+\Phi_{X\to Y_{3}}^{\lambda_{irr}}=\frac{-Theo:r_{0X}^{\lambda_{irr}}\;\left(or-Fit:r_{0X}^{\lambda_{irr}}\right)}{P_{0}^{\lambda_{irr}}\;\left(1-10^{-A_{X}^{\lambda_{irr}}\left(0\right)}\right)}.$$ (21)

The individual quantum yields of the divergent reaction steps ( $Y_{1}\;\leftarrow \;X\;\to \;Y_{3}$ ) can similarly be worked out [Eq. (22)] from their respective initial velocities ( $Fit:r_{0Y_{1}}^{\lambda_{irr}}$ and $Fit:r_{0Y_{3}}^{\lambda_{irr}}$ ).

$$\Phi_{X\to Y_{1\;or3}}^{\lambda_{irr}}=\frac{Fit:r_{0Y_{1\;or\;3}}^{\lambda_{irr}}}{P_{0}^{\lambda_{irr}}\;\left(1-10^{-A_{X}^{\lambda_{irr}}\left(0\right)}\right)}.$$ (22)

**FIGURE 8:**
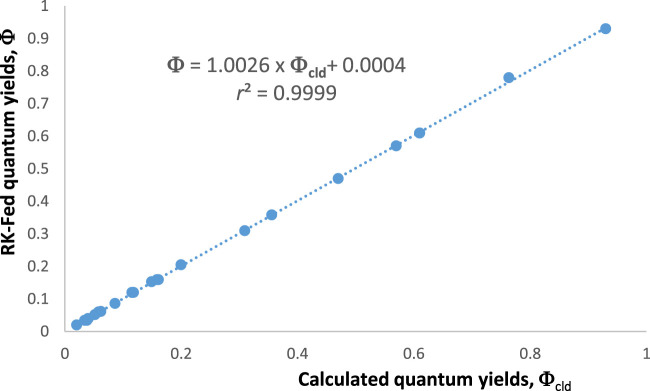
Excellent correspondence between the calculated quantum yields of X , $Y_{1}$ , and $Y_{3}$ using Eqs. 21, 22 (based on $Fit:r_{0X}^{\lambda_{irr}}$ values) and those feeding the RK calculations of these species’ traces.

Figure 8 shows the efficiency of Eqs. (21) and (22) in estimating the original quantum yield values that fed the RK-generated traces of species X , $Y_{1}$ , and $Y_{3}$ . Accordingly, the present photokinetic methodology of determining the quantum yields of the reactant and its immediate photoproducts is relatively easy and might be found beneficial to experimentalists including non-specialists of photokinetics.

### 3.11 Solving for all species’ individual quantum yields

Fully solving photokinetics of a given reaction would necessarily mean that all extrinsic and intrinsic parameter values are unequivocally determined. The extrinsic parameters are easier to obtain with relatively high accuracy. Intrinsic parameters’ values ( $\varepsilon_{Y_{j}}^{\lambda_{irr}}$ and $\Phi_{Y_{j}\to Y_{j^{\prime }}}^{\lambda_{irr}}$ ) are most often, all or in large majority, unknown to the investigator, with being relatively stringent to define with precision if at all definable. In fact, no methodology has ever been standardised to solve photokinetics irrespective of the reaction mechanism. There are, however, a few examples of working approaches addressing a handful reaction cases (Crano and Guglielmetti, 2003; Tonnesen, 2004; Pianowski, 2022). Only a fraction of those were based on photokinetic analysis. The approach presented in Section 3.10 is efficient but limited to the value of the quantum yield of X , failing to inform on the values of the rest of the intrinsic parameters of the reactive system. A more efficient approach, based on a semi-empirical method, readily solved the photokinetics of few reactions (Maafi and Maafi, 2013; Maafi and Lee, 2014a; Maafi and Lee, 2014b; Maafi and Lee, 2015a; Maafi and Lee, 2015a; Maafi and Lee, 2015b; Maafi and Lee, 2016).

The formalism presented hereafter claims, for the first time in the photochemistry literature, to provide a general methodology to extract the full set of intrinsic parameters characterising the reactive species of any photoreaction.

The step-by-step procedure is deployed as follows. Its application considers that i) the investigator knows precisely the mechanism operating the photoreaction, ii) the individual $C_{Y_{j}}^{\lambda_{irr}}\left(t\right)$ traces corresponding to each species of the reaction mechanism are experimentally collected. In addition, iii) the medium total absorbance $A_{tot}^{\lambda_{irr}}\left(t\right)$ trace is available, and iv) the values of the extrinsic parameters of the reaction ( $C_{X}^{\lambda_{irr}}\left(0\right)$ , $P_{0}^{\lambda_{irr}}$ , $l_{irr}$ , $l_{obs}$ , etc.) are all knowns. Conversely to points i) to iv), the procedure considers that the investigator v) has no indications relative to the values of the intrinsic parameters.

In the present study, we will use the RK-calculated traces but, while applying the solving method derived as follows, we will consider unknown intrinsic parameters’ values (that originally served to feed the RK calculation).

The general procedure for solving the kinetics for a given reaction mechanism, which may include one to eight species and $n_{\Phi }$ reaction steps (Scheme 1) subjected to a light beam of wavelength $\lambda_{irr}$ , unravels following three stages.

#### 3.11.1 Stage 0: the unknowns

For a reaction involving $n_{sp}$ species (including the reactant), and $n_{\Phi }$ reaction steps, the unknows are $n_{sp}$ absorption coefficients ( $\varepsilon_{Y_{j}}^{\lambda_{irr}}$ one per species at $\lambda_{irr}$ ) and $n_{\Phi }$ quantum yields ( $\Phi_{Y_{j}\to Y_{j^{\prime }}}^{\lambda_{irr}}$ one per reaction step). In total, there are $n_{sp}+n_{\Phi }$ unknowns to be determined. The values of extrinsic parameters are all supposed known. For simplicity, the trace for the total absorbance should be collected at $\lambda_{obs}=\lambda_{irr}$ and multiplied by ( $l_{irr}/l_{obs}$ ), such as $A_{tot}^{\lambda_{irr}}\left(t\right)=A_{tot}^{\lambda_{irr}/\lambda_{irr}}\left(t\right)=A_{tot}^{\lambda_{irr}/\lambda_{obs}}\left(t\right)\;l_{irr}/l_{obs}$ , in order to use $A_{tot}^{\lambda_{irr}}\left(t\right)$ in the following equations.

#### 3.11.2 Stage 1: determination of the fitting equations for the traces

(1) Fitting the $n_{sp}$ $C_{Y_{j}}^{\lambda_{irr}}\left(t\right)$ and the $A_{tot}^{\lambda_{irr}}\left(t\right)$ traces with adequate Eq. 4: The resulting $n_{sp}+1$ equations are recorded with the specific values for $\omega_{ij}^{\lambda_{irr}}$ , ${cc}^{\lambda_{irr}}$ , and $k_{ij}^{\lambda_{irr}}$ for each species $Y_{j}$ and the total absorbance.
(2) The graph showing the overlapping $C_{Y_{j}}^{\lambda_{irr}}\left(t\right)$ traces of the $n_{sp}$ species is drawn.

#### 3.11.3 Stage 2: determination of the $n_{sp}$ unknown absorption coefficients

(3) Values of the total absorbance are measured at $n_{\Phi }$ selected time intervals (selected from the traces plot constructed in point (**2**), using its fitting equation set out in point (**1**).
(4) Using the fitting equations obtained in point (**1**), the concentrations of each $Y_{j}$ species for $n_{\Phi }$ different time intervals selected in point (**3**) (a total of $n_{\Phi }\times n_{sp}$ concentration values, $n_{\Phi }\geq n_{sp}-1$ ) are worked out.
(5) Values of total absorbances and species concentrations in a set of corresponding $n_{sp}$ equations of the form given by Eq. (3) (chose $n_{sp}$ equations amongst the $n_{\Phi }\times n_{sp}$ possible). This will deliver $n_{sp}$ linear but linearly independent equations of $A_{tot}^{\lambda_{irr}}\left(t\right)$ .
6) Sole the system of these $n_{sp}$ linear equations for the $n_{sp}$ values of $\varepsilon_{Y_{j}}^{\lambda_{irr}}$

#### 3.11.4 Stage 3: determination of the $n_{\Phi }$ quantum yields of the individual reaction steps

(7) The individual $n_{\Phi }$ rate laws ( $r_{Y_{j}}^{\lambda_{irr}}\left(t\right)$ ) for the reaction species are written down according to Eq. (1) (there can be up to $n_{\Phi }\times n_{sp}$ different equations of $r_{Y_{j}}^{\lambda_{irr}}\left(t\right)$ that are written using $n_{\Phi }\times n_{sp}$ values of the species concentrations (**4**), total absorbances (**5**), and the $n_{sp}$ values of the species absorbance coefficients determined in point (**6**)). $n_{\Phi }$ equations of $r_{Y_{j}}^{\lambda_{irr}}\left(t\right)$ are selected for the rest of the treatment.
(8) The numerical values of the $n_{\Phi }$ $r_{j}^{\lambda_{irr}}\left(t\right)$ selected in point (**7**) are calculated, for the individual species $Y_{j}$ , as given by Eq. (5), using the values of $C_{Y_{j}}^{\lambda_{irr}}\left(t\right)$ obtained in point (**4**), the total absorbances values measured in point (**5**), and the absorbance coefficients derived in point (**6**).
(9) The same data described in point (**8**) is used to calculate the values of $A_{Y_{j}}^{\lambda_{irr}}\left(t\right)=\varepsilon_{Y_{j}}^{\lambda_{irr}}\;l_{irr}\;C_{Y_{j}}^{\lambda_{irr}}\left(t\right)$ at the $n_{\Phi }$ selected time intervals.
(10) The numerical values of $P_{a_{Y_{j}}}^{\lambda_{irr}}\left(t\right)$ at the $n_{\Phi }$ selected time intervals are calculated using Eq. 2 and the adequate values of $C_{Y_{j}}^{\lambda_{irr}}\left(t\right)$ (**4**), $A_{tot}^{\lambda_{irr}}\left(t\right)$ (**5**), $\varepsilon_{Y_{j}}^{\lambda_{irr}}$ (**6**), and $A_{Y_{j}}^{\lambda_{irr}}\left(t\right)$ (**9**).
(11) The aforementioned numerical values of $P_{a_{Y_{j}}}^{\lambda_{irr}}\left(t\right)$ (**10**) and $r_{Y_{j}}^{\lambda_{irr}}\left(t\right)$ (**8**) are introduced in $n_{\Phi }$ parametric rate-law equations (**7**) that include $n_{\Phi }$ unknown quantum yields.
(12) This system of $n_{\Phi }$ linear but linearly independent equations is solved for the $n_{\Phi }$ values of $\Phi_{Y_{j}}^{\lambda_{irr}}$

An illustration of the efficiency of the aforementioned method is provided in Table 1 for the photoreversible reaction (whose RK calculation was fed with $C_{X}^{\lambda_{irr}}\left(0\right)=1.58\;10^{-5}\;M$ , $P_{0}^{\lambda_{irr}}=1.25\;10^{-5}\;einstein\;s^{-1}\;dm^{-3}$ , $\varepsilon_{X}^{\lambda_{irr}}=12004\;M^{-1}\;cm^{-1}$ , $\varepsilon_{Y_{1}}^{\lambda_{irr}}=23123\;M^{-1}\;cm^{-1}$ , $\Phi_{X\to Y_{1}}^{\lambda_{irr}}=0.062$ , $\Phi_{Y_{1}\to X}^{\lambda_{irr}}=0.034$ , and $l_{irr}=1.65\;cm$ , assumed to be equal to $l_{obs}$ , and $\lambda_{irr}=\lambda_{obs}$ ). In this particular case, $n_{sp}=n_{\Phi }=2$ , which requires two equations of both $A_{tot}^{\lambda_{irr}}\left(t\right)$ and $r_{X}^{\lambda_{irr}}\left(t\right)$ for a complete elucidation of the kinetics (the former used for the extraction of the values of $\varepsilon_{X\;and\;Y_{1}}^{\lambda_{irr}}$ and the latter for those of $\Phi_{X\to Y_{1}}^{\lambda_{irr}}$ and $\Phi_{Y_{1}\to X}^{\lambda_{irr}}$ ).

**TABLE 1:** Data corresponding to the 12 points of the method solving the kinetics of a photoreversible reaction.

Point	Quantity	$n_{\Phi }\;time-intervals$	
		$t_{1}=30\;s$	$t_{2}=60\;s$
$\left(1\right)$	$C_{X}^{\lambda_{irr}}\left(t\right)=0.0649\;\mathrm{Log}\left(1+3.175\;10^{-5}\;e^{-0.09915\times t}\right)+0.492\;\mathrm{Log}\left(1+3.175\;10^{-5}\;e^{-0.04517\times t}\right)+8.1165\;10^{-6}$		
	$C_{Y_{1}}^{\lambda_{irr}}\left(t\right)=-0.06468\;\mathrm{Log}\left(1+3.175\;10^{-5}\;e^{-0.09915\times t}\right)-0.4922\;\mathrm{Log}\left(1+3.175\;10^{-5}\;e^{-0.04517\times t}\right)+7.6834\;10^{-6}$		
	$A_{tot}^{\lambda_{irr}}\left(t\right)=-1.942\;\mathrm{Log}\left(1+2.112\;10^{-2}\;e^{-0.09915\times t}\right)-13.58\;\mathrm{Log}\left(1+2.112\;10^{-2}\;e^{-0.04517\times t}\right)+0.45391$		
$\left(3\right)$	$A_{tot}^{\lambda_{irr}}\left(t\right)$	0.42096	0.44558
$\left(4\right)$	$C_{X}^{\lambda_{irr}}\left(t\right)$	$9.91197\;10^{-6}$	$8.57014\;10^{-6}$
	$C_{Y_{1}}^{\lambda_{irr}}\left(t\right)$	$5.88738\;10^{-6}$	7.22959 $10^{-6}$
$\left(5\right)$	$0.42096=9.91197\;10^{-6\times 1.65\times }\varepsilon_{X}^{\lambda_{irr}}+5.88738\;10^{-6\times 1.65\times }\varepsilon_{Y_{1}}^{\lambda_{irr}}$		
	$0.44558=8.57014\;10^{-6\times 1.65\times }\varepsilon_{X}^{\lambda_{irr}}+7.22959\;10^{-6\times 1.65\times }\varepsilon_{Y_{1}}^{\lambda_{irr}}$		
$\left(6\right)$	$\varepsilon_{X}^{\lambda_{irr}}=12006.43\;\left(\%\;Err^{*}=0.02\right)$		
	$\varepsilon_{Y_{1}}^{\lambda_{irr}}=23120.78\;\left(\%\;Err^{*}=0.01\right)$		
$\left(7\right)$	$r_{X}^{\lambda_{irr}}\left(t\right)=-\Phi_{X\to Y_{1}}^{\lambda_{irr}}\;P_{aX}^{\lambda_{irr}}\left(t\right)+\Phi_{Y_{1}\to X}^{\lambda_{irr}}\;P_{aY_{1}}^{\lambda_{irr}}\left(t\right)$		
	$r_{Y_{1}}^{\lambda_{irr}}\left(t\right)=-\Phi_{Y_{1}\to X}^{\lambda_{irr}}\;P_{aY_{1}}^{\lambda_{irr}}\left(t\right)+\Phi_{X\to Y_{1}}^{\lambda_{irr}}\;P_{aX}^{\lambda_{irr}}\left(t\right)$		
$\left(8\right)$	$r_{X}^{\lambda_{irr}}\left(t\right)$	$-8.35686\;10^{-8}$	–
	$r_{Y_{1}}^{\lambda_{irr}}\left(t\right)$	–	$2.06243\;10^{-8}$
$\left(9\right)$	$A_{X}^{\lambda_{irr}}\left(t\right)$	0.19636204	0.1697796
	$A_{Y_{1}}^{\lambda_{irr}}\left(t\right)$	0.22459924	0.2758036
$\left(10\right)$	$P_{aX}^{\lambda_{irr}}$	$3.61887\;10^{-6}$	$3.05565\;10^{-6}$
	$P_{aY_{1}}^{\lambda_{irr}}$	$4.13927\;10^{-6}$	$4.96385\;10^{-6}$
$\left(11\right)$ and $\left(12\right)$	$\Phi_{X\to Y_{1}}^{\lambda_{irr}}=0.06198\;\left(\%\;Err^{*}=0.031\right)$		
	$\Phi_{Y_{1}\to X}^{\lambda_{irr}}=0.03399\;\left(\%\;Err^{*}=0.002\right)$		

*: % Err is the percentage error of the obtained value to the value that fed the RK calculation.

Therefore, it is clear that the aforementioned procedure is able to solve for intrinsic parameters of any reaction photokinetics and, hence, definitely settles this, almost a century-old, problem.

### 3.12 On the generally adopted quantum yield formula

By considering the rate-law equation of a species $Y_{j}$ [as given by Eq. (1)], it is mathematically consistent to perform an integration of the two terms of the equation, independently from one another, when separation of the variables is feasible. Here, each term of the differential equation is integrated with respect to a different variable. For a rate law, this means that the left-hand side of the equation is integrated relative to the concentration whereas the right-hand-side term is integrated relative to time. However, this imposes that the right-hand-side term is a function of time [not absorbances as stated in Eq. (1)]. In other words, the concentrations making part of the absorbances should be replaced by Eq. 4 that are functions of time. Under these conditions, we can write the integral of the rate law in the following general form:

$$\int_{C_{j}^{\lambda_{irr}}\left(0\right)}^{C_{j}^{\lambda_{irr}}\left(t\right)}\;dC=\sum_{i=0}^{n_{\Phi j}}-\Phi_{Y_{j}\;\to \;Y_{i}}^{\lambda_{irr}}\;\int_{0}^{t}P_{a_{Y_{j}}}^{\lambda_{irr}}\left(t\right)\;dt+\Phi_{Y_{i}\;\to \;Y_{j}}^{\lambda_{irr}}\;\int_{0}^{t}P_{a_{Y_{i}}}^{\lambda_{irr}}\left(t\right)\;dt.$$ (23)

For the reactant, we can write its equation by also making, in the right-hand-side term, the coefficient corresponding to the first reaction step ( $X\;\to \;Y_{1}$ ) visible as

$$\int_{C_{X}^{\lambda_{irr}}\left(0\right)}^{C_{X}^{\lambda_{irr}}\left(t\right)}\;dC=-\Phi_{X\;\to \;Y_{1}}^{\lambda_{irr}}\int_{0}^{t}P_{a_{X}}^{\lambda_{irr}}\left(t\right)\;dt+\sum_{i=1}^{n_{\Phi j}-1}-\Phi_{X\;\to \;Y_{i}}^{\lambda_{irr}}\int_{0}^{t}P_{a_{X}}^{\lambda_{irr}}\left(t\right)\;dt+\Phi_{Y_{i}\;\to \;X}^{\lambda_{irr}}\int_{0}^{t}P_{a_{Y_{i}}}^{\lambda_{irr}}\left(t\right)\;dt.$$ (24)

Hence, we integrate the left-hand side and rearrange Eq. (24) so that it gives the expression for the initial quantum yield.

$$\Phi_{X\;\to \;Y_{1}}^{\lambda_{irr}}=-\frac{C_{X}^{\lambda_{irr}}\left(t\right)-C_{X}^{\lambda_{irr}}\left(0\right)}{\int_{0}^{t}P_{a_{X}}^{\lambda_{irr}}\left(t\right)\;dt}+\sum_{i=1}^{n_{\Phi j}-1}\left(-\Phi_{X\;\to \;Y_{i}}^{\lambda_{irr}}+\Phi_{Y_{i}\;\to \;X}^{\lambda_{irr}}\;\frac{\int_{0}^{t}P_{a_{Y_{i}}}^{\lambda_{irr}}\left(t\right)\;dt}{\int_{0}^{t}P_{a_{X}}^{\lambda_{irr}}\left(t\right)\;dt}\right).$$ (25)

Even though Eqs. (24) and (25) are valid (under the condition stated previously), and the integration of the left-hand term of Eq. (24) is straightforward, it is still difficult, in general, to obtain the values of the integrals of the absorbed light by the different species ( $P_{a_{Y_{i}}}^{\lambda_{irr}}\left(t\right)$ ). This stems from complex formulations of the absorbed light terms as functions of time, when introducing the concentration explicit formula (the corresponding Eq. 4). One then obtains functions that do not have known antiderivatives. Therefore, analytical integrations on the right-hand side of Eqs. (24) and (25) are impossible for all photoreactions but one. Indeed, Eq. (25) is analytically integrated only for the primary photoreaction with a transparent photoproduct ( $\varepsilon_{Y_{1}}^{\lambda_{irr}}=0$ ). For this particular reaction, Eq. (25) becomes

$$\Phi_{X\;\to \;Y_{1}}^{\lambda_{irr}}=-\frac{C_{X}^{\lambda_{irr}}\left(t\right)-C_{X}^{\lambda_{irr}}\left(0\right)}{\int_{0}^{t}P_{a_{X}}^{\lambda_{irr}}\left(t\right)\;dt}.$$ (26)

After introducing the explicit formula of the concentration of X previously analytically derived (Maafi and Brown, 2007) (which has an Eq. 4 formulation), into the expression of the absorbed light, we obtain

$$P_{a_{X}}^{\lambda_{irr}}\left(t\right)=P_{0}^{\lambda_{irr}}\;\left(1-\frac{1}{10^{A_{X}^{\lambda_{irr}}\left(t\right)}}\right)=P_{0}^{\lambda_{irr}}\;\left(1-\frac{1}{1+\left(10^{A_{X}^{\lambda_{irr}}\left(0\right)}-1\right)\;e^{-k_{X}^{\lambda_{irr}}\;t}}\right).$$ (27)

Integration of Eq. (27) relative to dt , and introducing the result in the denominator of Eq. (26), we obtain

$$\Phi_{X\;\to \;Y_{1}}^{\lambda_{irr}}=\frac{C_{X}^{\lambda_{irr}}\left(t\right)-C_{X}^{\lambda_{irr}}\left(0\right)}{\left(P_{0}^{\lambda_{irr}}/k_{X}^{\lambda_{irr}}\right)\;\left(A_{X}^{\lambda_{irr}}\left(0\right)\;\ln\left(10\right)-\ln\left(1+\left(10^{A_{X}^{\lambda_{irr}}\left(0\right)}-1\right)\;e^{-k_{X}^{\lambda_{irr}}\;t}\right)\right)}.$$ (28)

The values of $\Phi_{X\;\to \;Y_{1}}^{\lambda_{irr}}$ obtained from Eq. 28 and the fitting parameters of the reactant RK trace are quasi-equal to those originally feeding the RK calculation. Another confirmation of Eq. (28) is brought about by Eq.(29), a rearranged integrated rate law of X (Maafi and Brown, 2007), where its parameters take the values that fed the RK calculation. Despite the notable differences in the format between these two equations [Eqs (28) and (29)], the values they generate for $\Phi_{X\;\to \;Y_{1}}^{\lambda_{irr}}$ are exactly similar.

$$\Phi_{X\;\to \;Y_{1}}^{\lambda_{irr}}=\frac{-1}{\varepsilon_{X}^{\lambda_{irr}}P_{0}^{\lambda_{irr}}\;l_{irr}\ln\left(10\right)\;t}\;\ln\frac{\left(10^{A_{X}^{\lambda_{irr}}\left(t\right)}-1\right)}{\left(10^{A_{X}^{\lambda_{irr}}\left(0\right)}-1\right)}.$$ (29)

It is to be noted that when the photoproduct of the primary photoprocess absorbs ( $\varepsilon_{Y_{1}}^{\lambda_{irr}}\neq 0$ ), the integrals on the right-hand side of Eq. (24) [and Eq. (25)] become insolvable by a closed-form integration due to missing known antiderivative. This remains true even if the corresponding Eq. 4 is introduced to replace the concentration of the reactant in the integrals. Unfortunately, the same situation will be faced for virtually all remaining photoreactions. However, after incorporating the adequate Eq. 4 in the expression under the integral, the evaluation of $P_{a_{Y_{j}}}^{\lambda_{irr}}$ integrations occurring on the right-hand side of Eq. (24) or (25) can eventually be evaluated numerically. Incidentally, the equation proposed by the IUPAC (Braslavsky, 2007) has never been used for the estimation of the quantum yields of a photoreversible reaction [whereas Eq. (24) should apply].

The aforementioned analysis shows that the quantum yield equation proposed in the IUPAC report (Braslavsky, 2007), which is equivalent to Eq. (26), is applicable to a very specific situation, namely, a primary photoprocess with $\varepsilon_{Y_{1}}^{\lambda_{irr}}=0$ , but is not a general equation as might have been suggested in that document. Instead, the only general equation for the reactant is Eq. (24) [or Eq. (25)] for any reaction mechanism. It is also interesting to observe that the definition put forward by Warburg (1917), Warburg (1920), Warburg (1924), Warburg and Rump (1929), and Rubin and Braslavski (2010) cannot be consistent as it defines the quantum yield as derived by dividing the difference of concentrations ( $C_{X}^{\lambda_{irr}}\left(t\right)-C_{X}^{\lambda_{irr}}\left(0\right)$ ) by the total light absorbed by the studied system (i.e., $P_{a}^{\lambda_{irr}}=\sum P_{aY_{j}}^{\lambda_{irr}}$ ). Furthermore, it is evident that the formula of the quantum yield proposed by Allmand (1926) and Rubin and Braslavski (2010), which amended that of Warburg, is only valid for the simplest primary photoprocess (where $\varepsilon_{Y_{1}}^{\lambda_{irr}}=0$ ), since this is the only case where the $\Phi_{Y_{j}}^{\lambda_{irr}}$ is evaluated by the difference of concentrations of X at t=0 and t , dividing the amount of light absorbed by that specific species ( X ) (e.g., Eq. (25) suggests otherwise for X of other reactions). It might then be useful to recommend that the reference IUPAC document (Braslavsky, 2007) is amended with the aforementioned information.

### 3.13 On the reactant’s quantum yield variability with the irradiation wavelength

The absolute quantum yield value of a reaction step ( $\Phi_{Y_{j}\;\to \;Y_{j^{\prime }}}^{\lambda_{irr}}$ ) at $\lambda_{irr}$ is a specific feature of species $Y_{j}$ at that irradiation wavelength. The mathematical formalism adopted in photokinetics does not, *a priori*, impose conditions on the values of $\Phi_{Y_{j}\;\to \;Y_{j^{\prime }}}^{\lambda_{irr}}$ when $\lambda_{irr}$ is changed. The variation in $\Phi_{Y_{j}\;\to \;Y_{j^{\prime }}}^{\lambda_{irr}}$ with irradiation has been acknowledged in the IUPAC report, but no details were provided (Braslavsky, 2007). Our approach may provide a handy way to test whether $\Phi_{X\to \;Y_{j}}^{\lambda_{irr}}$ is variable with $\lambda_{irr}$ by employing the method described in Section 3.10.

Furthermore, for a reactant trace, the ratio of the initial reaction rates ( $Theo:r_{0X}^{\lambda_{irr}}$ or $Fit:r_{0X}^{\lambda_{irr}}$ ) measured at any two different irradiation wavelengths $\lambda_{irr_{1}}$ and $\lambda_{irr_{2}}$ , is to be equal to the ratio given by Eq. (30) (that is worked out from Eq.(12)), only and only if $\Phi_{X\to Y_{1}}^{\lambda_{irr}}$ and/or $\Phi_{X\to Y_{3}}^{\lambda_{irr}}$ are invariant with irradiation wavelength (each has the same value for $\lambda_{irr_{1}}$ and $\lambda_{irr_{2}}$ ). Otherwise, Eq. (30) qualitatively proves that $\Phi_{X\to \;Y_{j}}^{\lambda_{irr}}$ is $\lambda_{irr}$ -dependent (even if the actual absolute values of the individual quantum yields are not yet known). It is to be noted that the last term of the right-hand side of Eq. (30) can be computed from available experimental data.

$$\frac{r_{0X}^{\lambda_{irr_{1}}}}{r_{0X}^{\lambda_{irr}^{\prime }}}=\frac{-\left(\Phi_{X\to Y_{1}}^{\lambda_{irr_{1}}}+\Phi_{X\to Y_{3}}^{\lambda_{irr_{1}}}\right)}{-\left(\Phi_{X\to Y_{1}}^{\lambda_{irr_{2}}}+\Phi_{X\to Y_{3}}^{\lambda_{irr_{2}}}\right)}\;\frac{P_{0}^{\lambda_{irr_{1}}}\;\left(1-10^{-A_{X}^{\lambda_{irr_{1}}}\left(0\right)}\right)}{P_{0}^{\lambda_{irr_{2}}}\;\left(1-10^{-A_{X}^{\lambda_{irr_{2}}}\left(0\right)}\right)}=\frac{P_{0}^{\lambda_{irr_{1}}}}{P_{0}^{\lambda_{irr_{2}}}}\;\frac{1-10^{-A_{X}^{\lambda_{irr_{1}}}\left(0\right)}}{1-10^{-A_{X}^{\lambda_{irr_{2}}}\left(0\right)}}.$$ (30)

The simplicity of the method is an advantage to quickly test whether the quantum yield is constant with wavelength. This might be of interest to the community as there are not many evaluations of the invariability of the quantum yield with irradiation, probably considered irrelevant owing to the general assumption that such an invariability should be taken for granted. The argument behind such a state of the matter relates to the Kasha–Vavilov rule (Kasha, 1950), despite that this rule concerned fundamentally photophysical processes and has not been expanded, by the authors, to photochemistry. The literature has reported a large number of systems where the quantum yield is not constant over two or more wavelengths (Becker et al., 1969; Becker and Favaro, 2011; Reinfelds et al., 2019; Montalti et al., 2020), including the ferrioxalate actinometer (Montalti et al., 2020), since as early as 1958 (Zimmerman, 1958). In our team, using the semi-empirical method and by systematic screenings, we have observed the variability of quantum yields with $\lambda_{irr}$ for species involved in various mechanisms, whose reactants belonged to several chemical families, and their reactive systems have diverse applications. Linear, triangular, and sigmoid variations of $\Phi_{X\to \;Y_{1}}^{\lambda_{irr}}$ with $\lambda_{irr}$ have been observed (Maafi and Maafi, 2013; Maafi and Maafi, 2014a; Maafi and Maafi, 2014b; Maafi and Lee, 2015a; Maafi and Maafi, 2015a; Maafi and Lee, 2015b; Lee, 2016; Maafi and Maafi, 2016; Maafi and Al-Qarni, 2018; Maafi and Al-Qarni, 2019). It is then reasonable to consider the aforementioned results as a recurrent and objective experimental observation, which cannot be simply dismissed by invoking experimental discrepancies. In addition, up to date, no fundamental explanation has been proposed and accepted by the community for a supposed invariability of Φ with $\lambda_{irr}$ . The approach described above, might be able to help settle the debate in an effective and easy-to-implement way.

The methods and procedures presented in the previous sections prove a number of reaction behaviours and features and describe the ways to quantify them. However, one needs to keep in mind several important considerations. The approaches are built on the consideration that the concentrations of the reactive species of the investigated photoreaction, at any reaction time, all fall within the respective linearity ranges of their individual calibration graphs. Hence, it is highly recommended to experimentally apply these methods only to a system whose reactant initial concentration is relatively low (the lower, the better but, perhaps, where the total absorbance of the medium is, at least, below 0.5 at any reaction time. At such a value of the total absorbance, it might reasonably be assumed that, for a wide range of organic molecules, the concentrations of the species fall within their linearity ranges).

Knowing that the fitting performance of the experimental traces with Eq. 4 is tributary to both the experimental data quality and quantity, it is recommended to ensure such data have high precision.

## 4 Concluding remarks

Numerical integration (RK-NIM) has been used in the present work not for kinetic elucidation, as usually proposed in the literature, but for the purpose of describing the behaviours of photoreactions in different situations. Based on the initial velocity, as a metric, several reactivity features have been quantified and proven to generally occur for photoreactions, regardless of the governing mechanism.

A model equation, of the Φ -order character, has proven to faithfully reproduce the kinetic traces generated by RK-NIM. It stands for a unifying model able to describe photokinetics in the many reaction conditions and properties laid out here but expected also to be, in general, valid for other situations not described in the present work.

This model equation is a meaningful tool, as it facilitates full solving for the intrinsic parameters of photoreactions, quantifies the effects of various factors influencing reactivity, and delivers kinactinometers, in relatively handy procedures.

Overall, the findings of this work contribute to standardising photokinetic investigation and lay solid grounds for further developments in this important subject.

The application of a similar approach, to that developed here, is ongoing for the photokinetics under polychromatic light.

Furthermore, it is conjectured that the strategy presented here might be of interest to studying experimental setups of different reactor geometry and spatial distribution of the incidence radiation. The Φ -order kinetic character of photoreactions is expected to be preserved under those conditions, for which Eq. 4 template should apply.

## Figures and Tables

**Table:** 

$A_{tot}^{\lambda_{irr}}\left(t\right)$	Total absorbance of the reactive medium at wavelength $\lambda_{irr}$ and $l_{irr}$ at time t (also trace of the medium’s total absorbance). $A_{tot}^{\lambda_{irr}}\left(t\right)=A_{tot}^{\lambda_{irr}/\lambda_{irr}}\left(t\right)$
$A_{Y_{j\;or\;j^{\prime }}}^{\lambda_{irr}}\left(t\right)$	Absorbance of species $Y_{j}$ (or $Y_{j^{\prime }}$ ) measured at wavelength $\lambda_{irr}$ and $l_{irr}$ at time t (also trace of $Y_{j}$ (or $Y_{j^{\prime }}$ ) absorbance). $A_{Y_{j\;or\;j^{\prime }}}^{\lambda_{irr}}\left(t\right)=A_{Y_{j\;or\;j^{\prime }}}^{\lambda_{irr}/\lambda_{irr}}\left(t\right)$
$A_{tot}^{\lambda_{irr}/\lambda_{obs}}\left(t\right)$	Total absorbance of the reactive medium at time t irradiated at $\lambda_{irr}$ and observed at $\lambda_{obs}$ , where the optical path length is $l_{obs}$ (also trace of medium’s total absorbance)
$A_{tot}^{\lambda_{irr}/\lambda_{obs}}\left(\infty \right)$	Total absorbance of the reactive medium $A_{tot}^{\lambda_{irr}/\lambda_{obs}}\left(t\right)$ at the end of the reaction ( t=∞ )
$A_{{SPM}_{r}}^{\lambda_{irr}}$	Total absorbance of the spectator molecules present in the reactive medium ( 0≤r≤w )
c	Velocity of light given (in m/s )
$C_{X}^{\lambda_{irr}}\left(t\right)$	Kinetic traces of X ( $X=Y_{0}$ ), or the value of X concentration at time t (in M ), when the reaction is driven by a light beam of wavelength $\lambda_{irr}$
$C_{Y_{j}}^{\lambda_{irr}}\left(t\right)$	Kinetic traces of $Y_{j}$ ( j≠0 ) or the value of $Y_{j}$ concentration at time t when the reaction is driven by a light beam of wavelength $\lambda_{irr}$
$C_{\infty ,j}^{\lambda_{irr}}$	Concentration of species $Y_{j}$ ( $n_{sp}\geq j\geq 0$ ) at the end of the considered reaction under irradiation with a light of wavelength $\lambda_{irr}$
${cc}_{j}^{\lambda_{irr}}$ **or** ${cc}^{\lambda_{irr}}$	Pre-exponential factor in Eq. 4 of species $Y_{j}$
e	Exponential function
$E_{h\nu }^{\lambda_{irr}}$	The energy (in J ) carried by one photon of the radiation of wavelength $\lambda_{irr}$
$E_{mol\;h\nu }^{\lambda_{irr}}$	The energy (in $J\;einstein^{-1}$ ) carried by 1 mol of photons of wavelength $\lambda_{irr}$
$E_{sp-irr}$	Spectral irradiance (in $J\;s^{-1}\;{cm}^{-2}\;{nm}^{-1}$ ) measured by physical actinometry for the incident light of wavelength $\lambda_{irr}$
$\varepsilon_{Y_{j}}^{\lambda_{irr}}$	Absorption coefficient of species $Y_{j}$ at $\lambda_{irr}$ (in $M^{-1}\;{cm}^{-1}$ )
${flx}_{h\nu }^{\lambda_{irr}}$	Incident photons flux of the monochromatic light through a unit area (in $einstein\;s^{-1}\;{cm}^{-2}$ )
${flx}_{h\nu ,S_{irr}}^{\lambda_{irr}}$	Flux of monochromatic photons entering the reactor through an area $S_{irr}$ (in $einstein\;s^{-1}$ )
j	Index for the species $Y_{j}$ , ( $n_{sp}\geq j\geq 0$ )
$j^{\prime }$	Index for the species Y ( $Y_{j^{\prime }}$ ) with $j^{\prime }\neq j$
$i_{A}$	Number of mono- Φ -order terms in a given Eq. 8, used for absorbance traces
$i_{j}$	Number of mono- Φ -order terms in a given Eq. 4, used for concentration traces
h	Planck’s constant (in J s )
$k_{ij}$	Rate constant of the *i* ^*th*^ regime of species $Y_{j}$ , occurring in the exponential of Eq. 4 (in $s^{-1}$ )
**LMA**	Levenberg–Marquardt algorithm
ln	Natural logarithm
Log	Logarithm base 10
$l_{irr}$	Optical path length of the irradiation light from the lamp inside the reactor (in cm )
$l_{obs}$	Optical path length of the monitoring light from the spectrophotometer inside the reactor
$\lambda_{irr}$ ***or*** $\lambda_{irr}^{\prime }$	Non-isosbestic irradiation wavelength (in nm , the wavelength of the light beam driving the reaction which is different from the wavelengths of isosbestic points)
$\lambda_{isos}$	Isosbestic wavelength, at which the medium is exposed. It corresponds to the wavelength of one of the isosbestic points observed on the timely absorption spectra of the reaction
$\lambda_{obs}$	Observation wavelength, at which the medium is monitored ( $\lambda_{obs}$ might be different from $\lambda_{irr}$ )
**NIMs**	Numerical integration methods
$n_{sp}$	Total number of species in the reaction medium, $0\leq j\leq n_{sp}$ ( $n_{sp}=8$ for the mechanism in Scheme 1)
$n_{\Phi }$	Total number of the reaction steps in the considered mechanism ( $1\leq n_{\Phi }\leq 14$ for the mechanism in Scheme 1)
$n_{\Phi j}$	Photoreaction steps starting or ending at species $Y_{j}$ ( $1\leq n_{\Phi j}\leq n_{\Phi }$ )
$PKF\left(t\right)$	Photokinetic factor of the reaction at hand at time t
$P_{0}^{\lambda_{irr}}$	Incident radiation intensity from the lamp at $\lambda_{irr}$ , entering the reactor (it is a flux of photons per s per the sample’s irradiated surface $S_{irr}$ and volume $V_{irr}$ ). It is expressed in $einstein\;s^{-1}\;{dm}^{-3}$
$P_{a}^{\lambda_{irr}}\left(t\right)$	Total absorbed light by the reactive medium at time t and at wavelength $\lambda_{irr}$
$P_{a_{Y_{j\;or\;j^{\prime }}}}^{\lambda_{irr}}\left(t\right)$	Fraction of $P_{a}^{\lambda_{irr}}$ that is specifically absorbed by species $Y_{j}$ (or $Y_{j^{\prime }}$ ) at time t and at wavelength $\lambda_{irr}$
$\Phi_{Y_{j}\;\to \;Y_{j^{\prime }}}^{\lambda_{irr}}$	Quantum yield of the reaction step $Y_{j}\;\to \;Y_{j^{\prime }}$ ( $j\neq j^{\prime }$ )
$\Phi_{Y_{j^{\prime }}\;\to \;Y_{j}}^{\lambda_{irr}}$	Quantum yield of the reaction step $Y_{j^{\prime }}\;\to \;Y_{j}$ ( $j^{\prime }\neq j$ )
***r*** ^**2**^	Squared correlation coefficients
**RK**	Fourth-order Runge–Kutta numerical integration method (RK-NIM)
**RMSD**	Root mean square deviation
$r_{Y_{j}}^{\lambda_{irr}}\left(t\right)$	Rate law for species $Y_{j}$ , or $Y_{j}$ reaction-rate value at time t (in $M\;s^{-1}$ )
$r_{{0Y}_{j}}^{\lambda_{irr}}$	Initial reaction rate of species $Y_{j}$ ( $0\leq j\leq n_{sp}\;where\;Y_{0}=X$ )
$Fit:r_{{0Y}_{j}}^{\lambda_{irr}}$	Initial reaction rate of species $Y_{j}$ calculated by using trace fitting equation and parameters
$RK:r_{{0Y}_{j}}^{\lambda_{irr}}$	Initial reaction rate of species $Y_{j}$ calculated by RK-NIM
$Theo:r_{{0Y}_{j}}^{\lambda_{irr}}$	Initial reaction rate of species $Y_{j}$ calculated from the rate-law equation of $Y_{j}$
**SSE**	Sum of squares error
$S_{irr}$	Area of the sample (or reactor) that is under irradiation (in ${cm}^{2}$ )
$t_{1/2}$	Time by which half of the reactant has depleted (in s )
**VBA**	Visual basic applications
$V_{irr}$	Volume of the sample (or reactor) that is under irradiation (in ${dm}^{3}$ )
$w_{ij}$	Pre-logarithmic factor in Eq. 4 for the *i* ^*th*^ regime of species $Y_{j}$
X	Reactant $X=Y_{0}$
$Y_{j}$	Reactant ( j=0 ) or photoproducts ( j≠0 ), $0\leq j\leq n_{sp}$

## Data Availability

The original contributions presented in the study are included in the article; further inquiries can be directed to the corresponding author.
